# Targeting interventions for HIV testing and treatment uptake: An attitudinal and behavioural segmentation of men aged 20–34 in KwaZulu-Natal and Mpumalanga, South Africa

**DOI:** 10.1371/journal.pone.0247483

**Published:** 2021-03-10

**Authors:** James Bell, Sunny Sharma, Shawn Malone, Melissa Levy, Jemma Reast, Joanna Ciecieląg, Svetlana Gogolina, Tamara Ansons, Sanna Fourie, Ricardo Braz, Kristen Little, Nina Hasen

**Affiliations:** 1 Ipsos Healthcare, London, United Kingdom; 2 Population Services International, Johannesburg, South Africa; 3 Ipsos, Warsaw, Poland; 4 Global Science Organisation, Ipsos, London, United Kingdom; 5 Ask Afrika, Pretoria, South Africa; 6 Population Services International, Washington, DC, United States of America; RTI International, UNITED STATES

## Abstract

Despite recent improvements, men still have worse HIV outcomes than women in South Africa. This study describes how young men form distinct behavioural and attitudinal subgroups, and is intended to inform the design of targeted interventions to encourage HIV testing and initiation on antiretroviral therapy. Data were collected using a cross-sectional survey with questions on men’s attitudes, beliefs and behaviours around HIV/AIDS. A total of 2,019 men were randomly sampled from eight district municipalities in KwaZulu-Natal and Mpumalanga provinces between October 2018 and January 2019. Men were eligible to participate if they were aged 20–34, Black African, had an education level below university graduation, were aware of HIV and were willing to disclose whether they had tested for HIV. Each participant responded to a questionnaire asking about their demographic characteristics, reported sexual behaviour, engagement with HIV testing and treatment services, alcohol consumption, HIV knowledge, attitudes to gender equity and reported level of depressive symptoms. Data were analysed using canonical correlation, hierarchical clustering and factor analysis techniques to produce five groups of men. The results were synthesised using Human Centred Design principles to suggests areas for potential intervention for each segment. The results showed that men vary based on their attitudes to gender and masculinity, use of alcohol, testing and treatment behaviour, HIV-related fears and preferences for testing modalities. Segment 1 (21%) avoids the topic of HIV, perhaps fearful of the impact on his life. Segment 2 (23%) is well connected to his community and has social concerns about HIV. Segment 3 (15%) struggles with more distal determinants of HIV acquisition such as unemployment and poor mental health. Segment 4 (25%) has concerns about the lifestyle changes that would be required if he were HIV positive. Segment 5 (16%) has a strong traditional mindset and is fearful of the ramifications of HIV in his community. The results will be used to design targeted interventions to increase HIV testing and treatment rates among young men in South Africa. Further research is required to understand the impact of interventions designed in this way.

## Introduction

In South Africa men are less likely to know their HIV status, initiate treatment upon diagnosis or adhere to treatment compared to women [1–3]. Half of all HIV-related deaths in men are among those who have never sought HIV/AIDS care, and women are 27% less likely to die from HIV-related causes than men [4].

The literature describes difficulties men, and in particular young Black men in South Africa, face when engaging with HIV services. These include the influence of patriarchal modes of masculinity, the role of alcohol in decision-making, the legacy of childhood poverty and trauma, the pervasiveness of HIV stigma, worries about disclosure of their HIV status, and the lack of healthcare services specifically tailored to their needs [5–14]. Black South African men may also have challenges with employment and access to education which could reduce their capacity to engage with healthcare services [15, 16]. There is an increasing appreciation of the barriers that men face; however, strategies to increase men’s engagement with HIV services tend to take a “one-size-fits-all” approach.

In the private sector, “segmentations” (defined as a statistical method of classifying customers into groups based on their characteristics) are used to tailor products and services to sub-sections of the target market [17]. This method has been applied to understand online shopping habits, examine Airbnb usage preferences, market environmentally friendly wines and characterise consumer attitudes to animal welfare [18–21]. Segmentations may be based on demographic, geographic, psychographic or behavioural characteristics of the target population [22]. Despite difficulties in measuring the impact of these approaches, there is evidence to suggest that psycho-behavioural segmentations, which incorporate data on motivations, behaviours and beliefs, result in more homogeneous (and therefore recognisable and useful) segments than a purely demographic analysis [23–25].

In public health, segmentation has been applied to understand perceptions of HIV risk in Malawi, barriers to VMMC in Zambia and Zimbabwe, and needs and preferences for family planning technology in Niger [26–28]. However, it has been noted that these methods are used relatively rarely to target health interventions [22, 25].

It has also been noted that health interventions often do not adequately understand the needs of the communities for which they are intended [29]. Human Centred Design (HCD) approaches have been proposed as a potential solution [30]. While methods vary, HCD approaches are grounded on designing services or interventions based on the population’s needs, desires and experiences rather than those of the implementer, and in making those services/interventions more user-friendly [31–35]. HCD processes have been applied in public health settings, for example for chronic disease prevention, TB and HIV care and handwashing interventions [31, 36–38]. Evidence from formative qualitative research suggested that an HCD intervention may be appropriate in this context as men often perceive HIV services and unwelcoming and not designed with their needs in mind [14].

The aim of this study was to use a psycho-behavioural segmentation to define subgroups of young men in KwaZulu-Natal (KZN) and Mpumalanga (MPU), South Africa, based on their attitudes, beliefs, behaviours and needs in relation to HIV testing and treatment. The results were then incorporated into a human centred design (HCD) process to synthesize the salient characteristics of each segment and to develop hypotheses about their unmet needs to prepare for subsequent intervention prototyping, iteration and implementation phases.

## Methods

### Data collection

Data were collected using a questionnaire informed by a literature review and the results of a formative qualitative study [14]. The questionnaire is given in the S1 Questionnaire. Identified drivers and barriers to HIV testing and ARV linkage were coded according to the Theoretical Domain Framework (TDF) which systematically categorised the relevant attitudes, behaviours and beliefs to be measured in the questionnaire [39].

The questionnaire collected demographic data, reported sexual behaviour, engagement with HIV testing and treatment services, alcohol consumption, HIV knowledge, attitudes to gender equity using the Gender-Equitable Men Scale (GEMS) and a measure of depression using the Patient Health Questionnaire (PHQ-9), which have both been tested and implemented in South Africa [40–46]. Respondents also indicated their agreement with a series of attitude statements on a 5-point Likert scale, which were written to measure men’s attitudes to the relevant domains of the TDF. The questionnaire was translated into isiZulu, Sepedi, SiSwati and Xitsonga and data were collected by trained enumerators, of all of whom were male residents of KZN or MPU, using Computer Assisted Personal Interviewing (CAPI) devices. Interviewer training took place over four days, which included at least one pilot interview for each interviewer, conducted among the communities around the training venues in Durban, KZN and Middleburg, MPU.

The survey was administered in all district municipalities of MPU (Ehlanzeni, Gert Sibande and Nkangala) and five district municipalities of KZN (eThekwini, King Cetshwayo, Ugu, uMgungundlovu and Zululand) between October 2018 and January 2019. The questionnaire was completed either in the respondent’s home, or in another place of their choosing (such as outside of the house for greater privacy). All responses (including HIV status and engagement in treatment) were self-reported and not otherwise verified. The districts in KZN were selected non-randomly in consultation with health authorities to prioritise areas with high HIV prevalence or areas which were considered high priority for intervention to improve HIV testing and treatment.

Participants were eligible if they resided in the districts of interest, were male, aged 20–34, Black African, had an education level below university graduation, were aware of HIV, and were willing to disclose whether they had been tested for HIV. Men were included in the study regardless of whether they had been tested for HIV or knew the result of the test. Men who had been medically circumcised after the age of 16 were limited to 20 percent of the sample to focus on those who had demonstrated lower engagement with the health system and were biomedically at greater risk of HIV. This cut-off point aimed to approximately match the prevalence of voluntary medical male circumcision (VMMC) in each of the two provinces and was decided on after a review of prevalence estimates from the two provinces. These ranged from 11.5% to 31.8% in MPU and 12.1% to 37.2% in KZN, leading to an approximate average of 20% across both provinces [47, 48]. Sampling was limited to low socioeconomic areas (defined as categories 1–4 of the Neighbourhood Lifestyle Index) to focus on men with limited access to health services [49].

The sampling frame was the South African Enumerated Area Frame (EAF), which was used to draw a random sample of sub-districts using probability proportional to size (PPS) methods, a multistage, stratified sampling methodology which was chosen to ensure that an adequate sample was obtained across urban and rural areas [49]. Within each subdistrict, enumeration areas (EAs) were stratified by EA type (urban, traditional and farm) and randomly selected according to PPS. Visiting points (corresponding to dwelling units or exact GPS coordinates in areas of informal housing) were randomly selected using maps produced in advance of fieldwork. Kish grids were used to select a dwelling if more than one was present on the visiting point, to select a household if more than one was present in the dwelling, and to select the respondent if more than one eligible man was present in the household. Three call-backs were attempted before a replacement household was selected using a random walk substitution method, which allowed for selection of another household close to the original target.

Written informed consent was obtained from all participants, and an honorarium was provided (ZAR 70 / USD 4.62). The research received ethical approval from the Population Services International Research Ethics Board in the USA and the Foundation for Professional Development Research Ethics Committee in South Africa. Approval was granted from the Department of Health in all districts and both provinces.

### Statistical analysis

The dataset used for analysis is given in the S1 Dataset. The segmentation analysis broadly followed the protocol detailed by Sgaier et al [27], which details an approach for dividing a sample into distinct attitudinal and behavioural groups. It involves a data reduction process based on canonical correlation, and a grouping process based on hierarchical cluster and k-means analysis, which groups respondents based on commonalities across the canonical variables [50, 51].

The study team selected variables (referred to as “segmentation variables”), based on the literature review and the formative qualitative study, which determined the constructs on which segments would be differentiated. A full list of these variables is given in the S1 Table. The number of segmentation variables was reduced using canonical correlation (to exclude those which showed little differentiation across respondents, or those which were highly correlated with other variables under consideration) and hierarchical clustering was used to develop initial cluster centres, which were then refined using k-means clustering to explore possible partitions to form segments [50, 51]. Several configurations of clusters were tested (with three, four and five segments), and the chosen solution was selected based on its ease of understanding and practical usability. The segments in the chosen solution were then profiled on the segmentation variables, and other demographic or attitudinal variables of interest (see Table 2). To aid interpretation, factor analysis was used to create aggregated measures of differentiation.

The figures presented are sample statistics and have not been weighted to population data in the two provinces. Differences between the segment means and proportions and total mean or proportion for each of the profiling variables were tested using T-tests. T-tests were used to test the null hypothesis that each segment’s factor score was equal to 0. Discriminant analysis was used to develop a “typing tool”–a short questionnaire to predict which segment a man falls into using a much shorter list of questions [52, 53]. All analyses were performed using SAS v9.4 and SPSS v24.

### Human centred design process

A workshop attended by a total of approximately twenty individuals, including the study team, the funder, representatives of a South African HCD agency, sector specialists from Population Services International (PSI) and experts in the HIV sector in South Africa) was convened in Johannesburg in February 2019. The goal of this meeting was to feed the research results into an intervention design process grounded in HCD principles. Specifically, this entailed synthesising the quantitative segmentation with the findings of formative qualitative research to produce short descriptions of each segment that identified their salient characteristics and attitudes and beliefs around HIV. The participants also produced a set of hypothesized intervention domains for each segment which formed the basis of the design, iteration and implementation of HCD-based interventions, largely following the approach from Ideo’s Design Kit [54]. Several methods were used including brainstorming, ‘how might we’ questions, roleplay, visualisation, theme identification and exercises to identify the relative priority of interventions for each segment. The process was led and moderated by the South African HCD company, with input of research findings by the study team. Active discussion according to a pre-agreed agenda was encouraged, and notes were taken by the research team throughout the workshop, which were synthesized into a summary report.

## Results

Between October 2018 and January 2019, 2,019 men were interviewed, 1,196 (59.2%) from KZN and 823 (40.8%) from MPU. 40.1% were aged 20–24, 37.2% aged 25–29 and 22.7% aged 30–34 (Table 1). 13.7% reported that they were HIV positive, 38.4% were uncircumcised.

**Table 1:** Demographic characteristics of the sample.

		Total		KwaZulu-Natal		Mpumalanga		P-value (difference between province proportions)
		Count	Column %	Count	Column %	Count	Column %	
**Age group**	20–24	809	40.1%	355	29.7%	454	55.2%	<0.001
	25–29	752	37.2%	479	40.1%	273	33.2%	0.02
	30–34	458	22.7%	362	30.3%	96	11.7%	<0.001
**HIV status**	Positive	276	13.7%	219	18.3%	57	6.9%	<0.001
	Negative	1511	74.8%	861	72.0%	650	79.0%	<0.001
	Never tested / Did not receive the results	232	11.5%	116	9.7%	116	14.1%	0.02
**Circumcision status**	Not circumcised	775	38.4%	562	47.0%	213	25.9%	<0.001
	Circumcised traditionally	331	16.4%	64	5.4%	267	32.4%	<0.001
	Circumcised in a clinic before the age of 16	530	26.3%	328	27.4%	202	24.5%	0.15
	Circumcised in a clinic after the age of 16	383	19.0%	242	20.2%	141	17.1%	0.08
**Highest level of education**	No formal schooling or qualifications	84	4.2%	35	2.9%	49	6.0%	0.01
	Some schooling but did not complete high school	571	28.3%	278	23.2%	293	35.6%	<0.001
	Finished high school	1209	59.9%	797	66.6%	412	50.1%	<0.001
	College/ vocational training	131	6.5%	75	6.3%	56	6.8%	0.63
	University [Not graduated]	24	1.2%	11	0.9%	13	1.6%	0.18

Unweighted sample results

### Segment descriptions

The segmentation produced five segments: Segment 1 (21.0%), Segment 2 (22.7%), Segment 3 (15.1%), Segment 4 (25.1%) and Segment 5 (16.2%) (Table 2).

**Table 2:** Segment characteristics.

		Total (n = 2019, 100%)		Segment 1 (n = 423, 21.0%)		Segment 2 (n = 458, 22.7%)		Segment 3 (n = 304, 15.1%)		Segment 4 (n = 507, 25.1%)		Segment 5 (n = 327, 16.2%)	
		Count	Column %	Count	Column %	Count	Column %	Count	Column %	Count	Column %	Count	Column %
**Province**	**KZN**	1196	59.2%	334	79.0%***	238	52.0%**	179	58.9%	242	47.7%***	203	62.1%
	**MPU**	823	40.8%	89	21.0%***	220	48.0%**	125	41.1%	265	52.3%***	124	37.9%
**Age**	**20–24**	809	40.1%	123	29.1%***	205	44.8%	119	39.1%	221	43.6%	141	43.1%
	**25–29**	752	37.2%	181	42.8%*	152	33.2%	109	35.9%	199	39.3%	111	33.9%
	**30–34**	458	22.7%	119	28.1%*	101	22.1%	76	25.0%	87	17.2%**	75	22.9%
**Highest level of schooling**	**No formal schooling or qualifications**	84	4.2%	15	3.5%	14	3.1%	23	7.6%**	18	3.6%	14	4.3%
	**Some schooling but did not complete high school**	571	28.3%	72	17.0%***	144	31.4%	96	31.6%	146	28.8%	113	34.6%*
	**Finished high school**	1209	59.9%	306	72.3%***	261	57.0%	159	52.3%*	304	60.0%	179	54.7%
	**College/ vocational training**	131	6.5%	28	6.6%	32	7.0%	22	7.2%	32	6.3%	17	5.2%
	**University [not graduated]**	24	1.2%	2	0.5%	7	1.5%	4	1.3%	7	1.4%	4	1.2%
**HIV status**	**Positive**	276	13.7%	54	12.8%	64	14.0%	51	16.8%	66	13.0%	41	12.5%
	**Negative**	1511	74.8%	327	77.3%	352	76.9%	208	68.4%*	374	73.8%	250	76.5%
	**Never tested / Did not receive the results**	232	11.5%	42	9.9%	42	9.2%	45	14.8%	67	13.2%	36	11.0%
**Circumcision status**	**Not circumcised**	775	38.4%	186	44.0%*	145	31.7%**	137	45.1%*	203	40.0%	104	31.8%*
	**Circumcised traditionally**	331	16.4%	53	12.5%*	80	17.5%	61	20.1%	89	17.6%	48	14.7%
	**Circumcised in a clinic before the age of 16**	530	26.3%	107	25.3%	122	26.6%	63	20.7%*	132	26.0%	106	32.4%*
	**Circumcised in a clinic after the age of 16**	383	19.0%	77	18.2%	111	24.2%*	43	14.1%*	83	16.4%	69	21.1%
**Visited a health clinic in the last 12 months**		1298	64.3%	286	67.6%	321	70.1%*	186	61.2%	320	63.1%	185	56.6%**
**Tested for HIV in the last year**		1223	67.6%	276	71.5%	277	66.3%	198	74.4%*	305	68.7%	167	56.8%***
**Ever taken ARVs [HIV+ only]**		202	75.1%	40	74.1%	49	81.7%	33	67.3%	45	69.2%	35	85.4%
**Always used as condom in the last year**		614	31.6%	126	30.9%	146	33.2%	53	18.7%***	141	28.5%	148	46.4%***
**Have a steady job**		710	35.2%	172	40.7%*	144	31.4%	105	34.5%	182	35.9%	107	32.7%
**Experience of moderate to severe depression**		177	8.8%	6	1.4%***	41	9.0%	89	29.3%***	25	4.9%**	16	4.9%*
**Drink alcohol every day**		84	4.2%	10	2.4%	16	3.5%	29	9.5%***	17	3.4%	12	3.7%
**Sometimes drink so much alcohol they don’t remember what happened**		721	35.7%	175	41.4%*	127	27.7%**	130	42.8%*	175	34.5%	114	34.9%
**Believe they have an important role to play in the community**		1452	71.9%	313	74.0%	390	85.2%***	177	58.2%***	341	67.3%*	231	70.6%
**Like the community in which they live**		1636	81.0%	332	78.5%	407	88.9%***	212	69.7%***	431	85.0%*	254	77.7%
**Mean number of sexual partners in the last 12 months**		2.3		2*		2.4		2.3		2.4		2.5	
**Mean HIV knowledge score**		6.4		7.0**		7.2***		2.6***		8.6***		4.7***	

Unweighted sample results.

* P<0.05,

** P<0.01,

*** P<0.001.

Segment values tested against sample total

#### Segment 1

**Fig 1:**
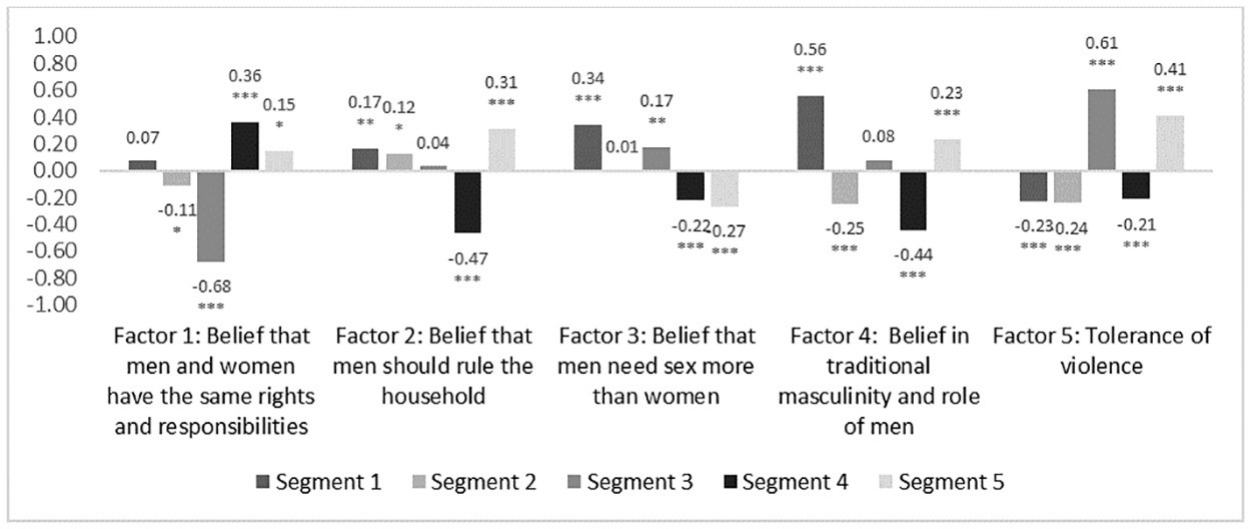
Attitudes towards relationships between men and women across derived factors by segment. * P<0.05, ** P<0.01, *** P<0.001. Segment values tested against sample total (standardised to 0). The factors scores were standardized with a mean of 0, meaning a bar above the x-axis represents a higher level of identification with that construct among that segment than average, and bars below the line represent a lower level of identification than average.

**Fig 2:**
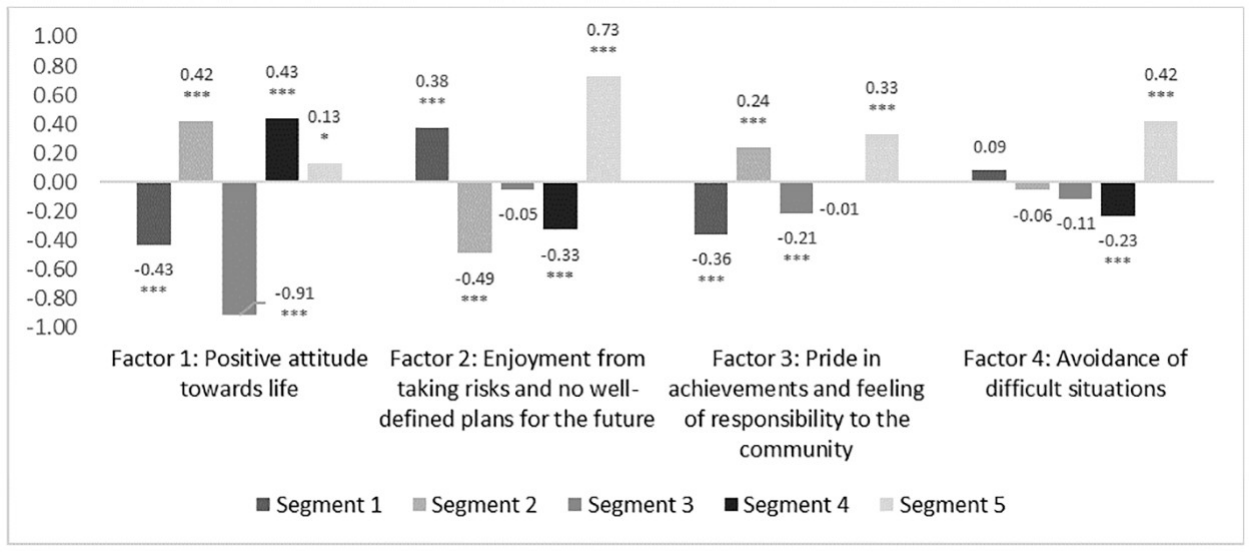
Attitudes to decision making and outlook on life across derived factors by segment. * P<0.05, ** P<0.01, *** P<0.001, Segment values tested against sample total (standardised to 0). The factors scores were standardized with a mean of 0, meaning a bar above the x-axis represents a higher level of identification with that construct among that segment than average, and bars below the line represent a lower level of identification than average.

Men in Segment 1 were among the most educated (72.3% finished high school in Segment 1 vs. 59.9% of total sample, p<0.001) and were most likely to have regular employment (40.7% vs. 35.2%, p = 0.03). They were also more likely to live in KZN (79.0% vs. 59.2%, p<0.001). They tended to hold traditional gender beliefs, agreeing men should make household decisions (factor score of 0.17, p = 0.02) and believing in traditional masculinity more than other segments (0.56, p<0.001) (Fig 1). They had a pessimistic outlook on life (-0.43 on positive attitude factor, p<0.001) and showed an inclination for risk-taking without well-defined plans for the future (0.38, p<0.001) (Fig 2).

**Fig 3:**
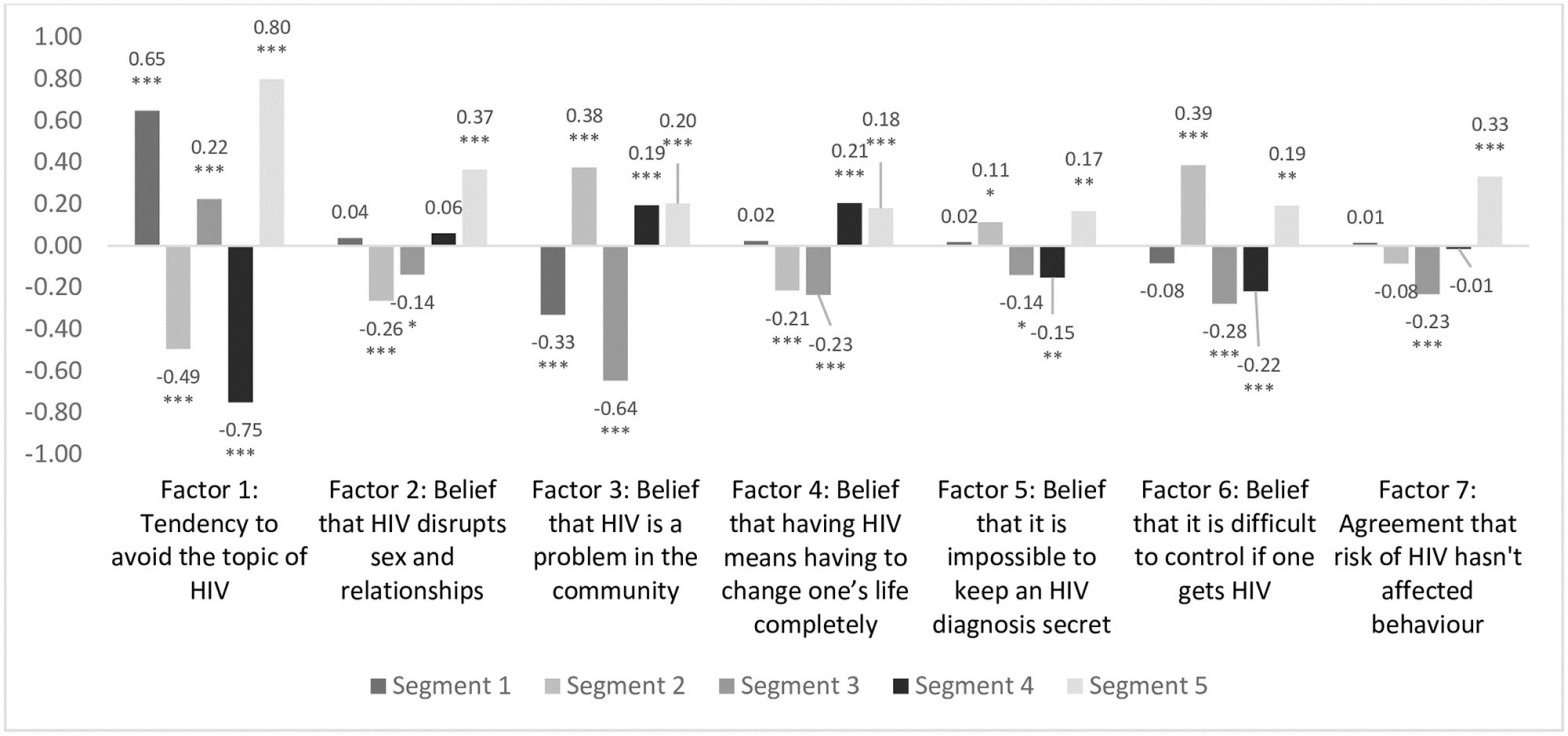
Attitudes to HIV across derived factors by segment. * P<0.05, ** P<0.01, *** P<0.001. Segment values tested against sample total (standardised to 0). The factors scores were standardized with a mean of 0, meaning a bar above the x-axis represents a higher level of identification with that construct among that segment than average, and bars below the line represent a lower level of identification than average.

This group was more likely to avoid the topic of HIV (0.65, p<0.001) and to believe that it was not a problem in their community (-0.33, p<0.001). They had comparatively higher knowledge of HIV (mean score of 7 vs 6.4, p<0.01) (Fig 3).

**Fig 4:**
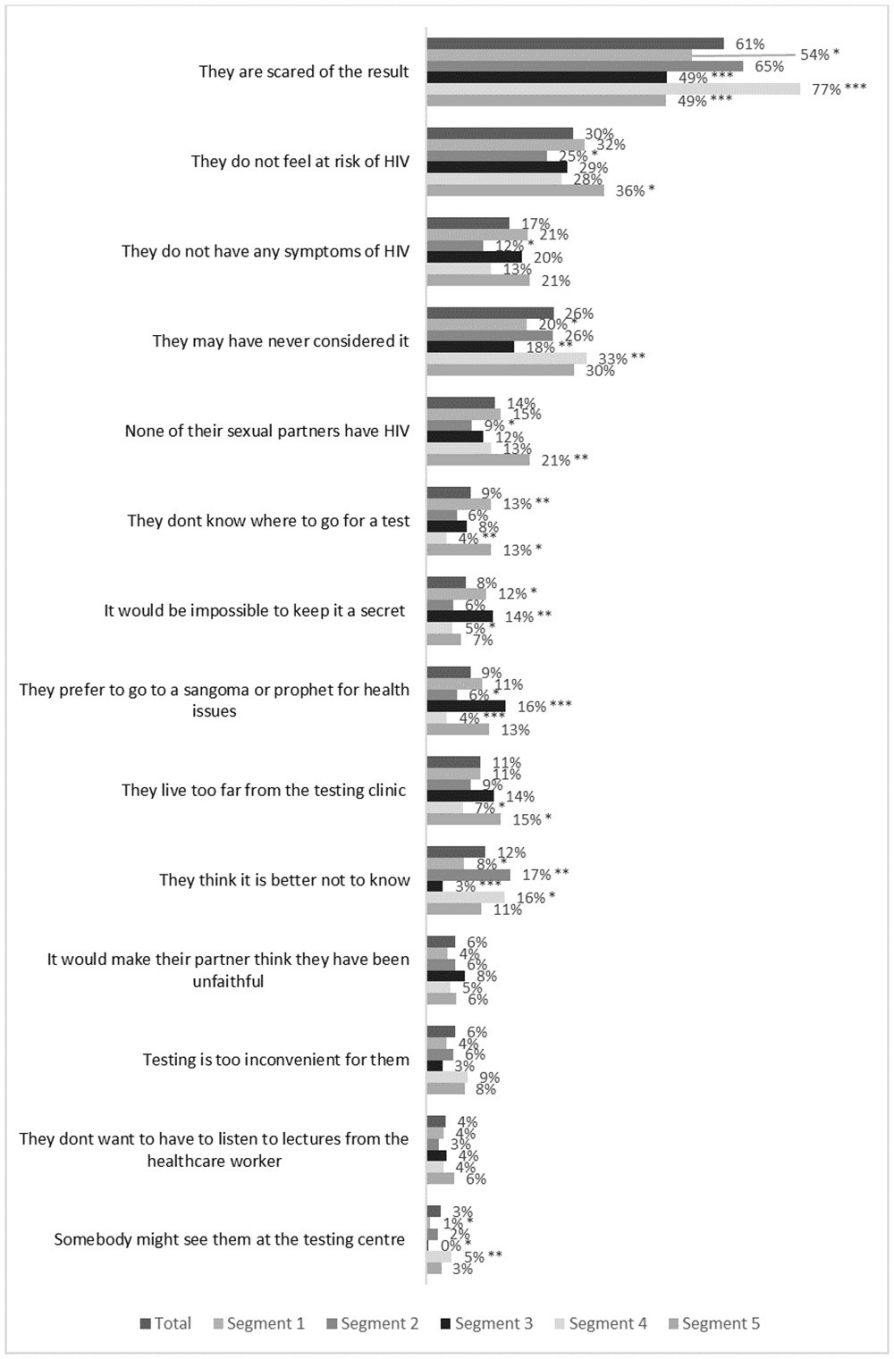
Reasons why respondents in each segment believe that other men do not test for HIV. Unweighted sample results, n = 1808 (respondents who have ever tested for HIV). * P<0.05, ** P<0.01 *** P<0.001. Segment values tested against sample total.

**Fig 5:**
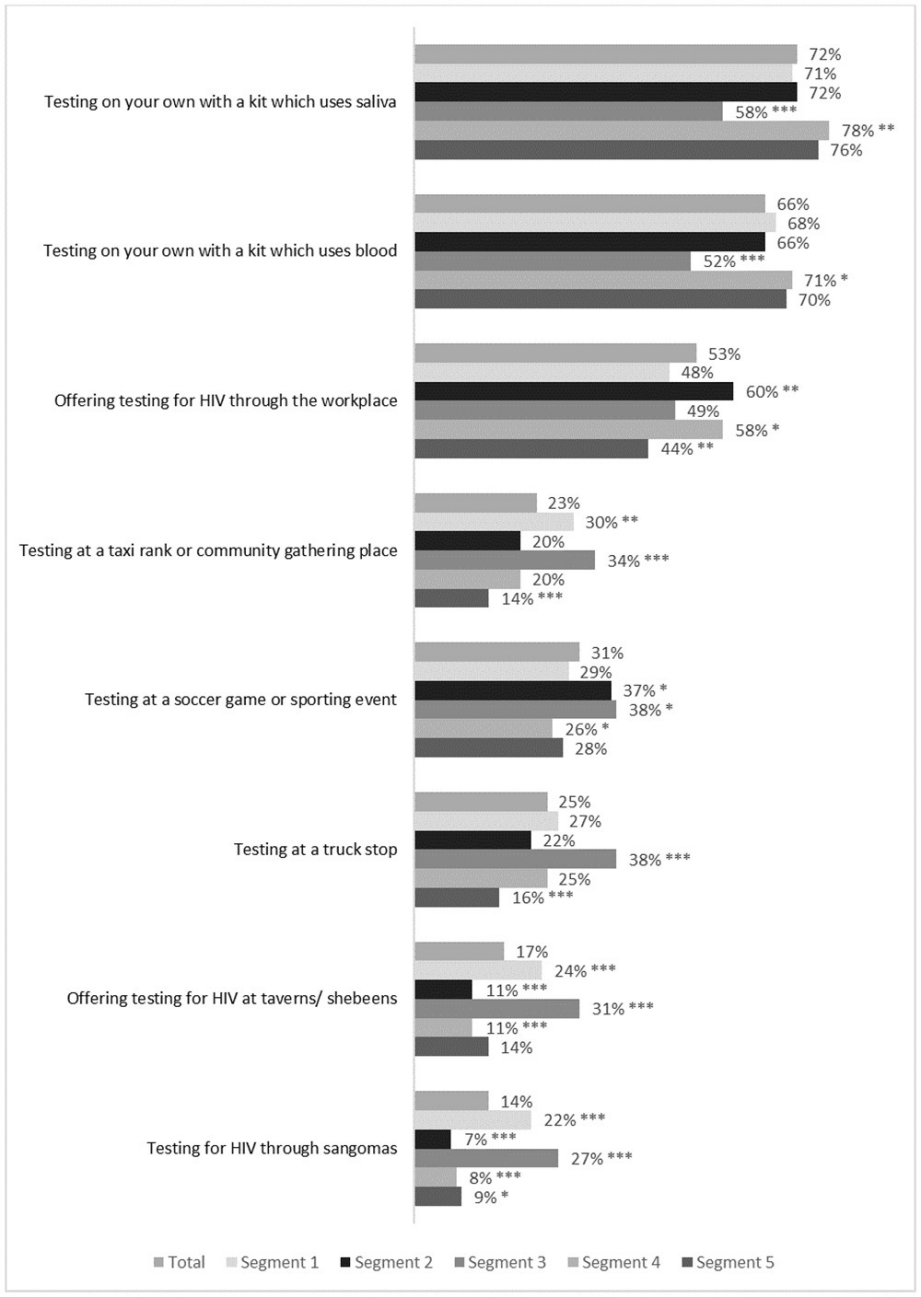
Percentage of respondents in each segment who believe that each service delivery option is a good idea or a very good idea. Unweighted sample results, n = 2019. * P<0.05, ** P<0.01, *** P<0.001 Segment values tested against sample total.

Segment 1 reported practical barriers to accessing care (13% said not knowing where to test is a barrier for others vs. 9% of the total sample, p<0.01) and greater fear that it would not be possible to keep the result a secret (12% vs 8%, p = 0.02) (Fig 4). They were less likely to cite fear as a reason for not testing (54% vs 61%, p = 0.02) or to think it is better not to know (8% vs 12%, p = 0.02). They were more likely than average to prefer testing at taxi ranks or community gathering places (30% vs 23%, p = 0.04), in taverns or shebeens (24% vs. 17%, p<0.001) or through sangomas (traditional healers) (22% vs. 14%, p<0.001) (Fig 5).

The HCD process determined that men in Segment 1 were relatively older, more educated and more financially stable, but with a more pessimistic outlook, lower sense of community belonging, higher alcohol consumption and a more negative view of the health system. Men in this segment visited clinics and tested for HIV reasonably often, but wished to avoid the topic as much as possible. They didnt believe that HIV would destroy their life, but it could further erode their sense of self-worth. Interventions focussed on the positive ramifications of HIV diagnosis and treatment in terms of quality of life and relationships with others could be appropriate for this group. See Table 3 for a summary of intervention design considerations from the HCD process.

**Table 3:** Summary of considerations for intervention design by segment.

Segment	Considerations for Intervention Design
One	• Focus on the positive outcomes of an HIV diagnosis
	• This could be, for example, improvements in quality of life or relationships
Two	• Disclosure should be addressed as a high priority
	• Could also introduce positive role models, e.g. people who live well with HIV
Three	• May be most worthwhile to target more distal determinants of HIV acquisition
	• This group may benefit from mental health support, employment guidance or better financial inclusion
	• May also be receptive to testing in public places such as truck stops or tax ranks, or with the help of traditional healers
Four	• Messaging could focus on the idea that people can live well with HIV and that diagnosis does not necessarily entail a complete change in lifestyle
	• U = U (undetectable = untransmissible) may resonate well
Five	• Messaging could emphasise the benefits of knowing one’s status for future health and for the good of one’s family

#### Segment 2

Segment 2 were more likely to live in MPU (48.0% in Segment 2 vs 40.8% in total sample, p<0.01). Men in Segment 2 were more likely to enjoy living in their community (88.9% vs. 81.0%, p<0.001). They were less likely to report a problematic relationship with alcohol (27.7% report drinking to the point of memory loss vs 35.7% in the total sample, p = 0.01). These men had more progressive attitudes towards gender relations (factor scores of -0.24 for belief in use of violence in relationships, p<0.001; -0.25 for belief in traditional masculinity, p<0.001), and a more positive attitude towards life and the future (0.42, p<0.001), combined with lower likelihood to take risks (-0.49, p<0.001).

70.1% said that they had visited a clinic in the last year, the highest of all segments (average of 64.3%, p = 0.02). Segment 2 had high HIV knowledge (mean score 7.2 vs 6.4 average, p<0.001). Although they were less likely than other segments to believe that HIV disrupts sex and relationships (factor score-0.26, p<0.001) or that a diagnosis would mean a complete change in lifestyle (-0.21, p<0.001) they were more likely to believe that it is a big problem in the community (0.38, p<0.001) and that is impossible to keep an HIV diagnosis secret from others (0.11, p = 0.03). They showed a preference for workplace testing (60% vs 53%, p = 0.004).

The HCD summary concluded that a man in Segment 2 tends to have a strong sense of connection to his community and is proud of the role he has there. Men in segment 2 have progressive ideas in terms of gender roles and don’t tend to drink to excess. While they have high HIV knowledge, they have concerns about what a positive diagnosis would mean for their standing in society. Interventions focussed on disclosure, potentially integrating positive role models who openly live with HIV for him to emulate, could be appropriate for Segment 2.

#### Segment 3

Men in Segment 3 had the lowest level of educational attainment (52.3% finished high school vs. average of 59.9%, p = 0.01). Segment 3 were among the least likely to say they liked the community where they live (69.7% vs. 81.0%, p<0.001)). 29.3% showed moderate to severe levels of depression (vs. average of 8.8%, p<0.001). They reported high frequency of alcohol consumption to the point of memory loss (42.8% vs. 35.7%, p = 0.02), and a pessimistic view of life (factor score on positive attitude towards life -0.91, p<0.001). Segment 3 also had the highest belief in use of violence in a relationship (0.61, p<0.001).

This segment was the least likely to report consistent condom use (18.7% always used in the last year vs. average of 31.6%, p<0.001) and had the lowest level of HIV knowledge (mean score of 2.6 vs. average of 6.4, p<0.001). This group’s attitudes towards HIV were characterised by avoidance (factor score 0.22, p<0.001).

This group was more likely to accept testing at community gathering places (34% vs. 23%, p<0.001), truck stops (38% vs. 25%, p<0.001) or via sangomas (27% vs 14%, p<0.001).

The HCD analysis found that men in Segment 3 tended to have a more pessimistic outlook on the future, and high levels of depression. They also have a traditional view of sex, gender and relationships and drink frequently. These men were very afraid of HIV but don’t know much about it. Interventions for this segment may be best targeted towards more distal determinants of health and HIV status such as mental health, employment or financial inclusion. The data also suggest that they are receptive to testing in public places such as truck stops or taxi ranks, or through traditional healers.

#### Segment 4

Segment 4 had low levels of depression (4.9% vs 8.8%, p<0.01) and a positive attitude to life (factor score 0.43, p<0.001). They had a more equitable view of the relationship between men and women (factor score for belief that men and women have the same rights and responsibilities: 0.36, p<0.001) and were more likely to reject traditional concepts of masculinity (-0.44, p<0.001).

Segment 4 had the highest knowledge of HIV across the segments (mean score 8.6 vs. 6.4, p<0.001). However, this segment was no more likely than average to have tested for HIV in the last year (68.7% vs 67.6%, p = 0.67), or to have begun taking ARVs after a positive diagnosis (69.2% vs. 75.1%, p = 0.33). This may be linked to the belief that a positive diagnosis would significantly disrupt his life (0.21, p<0.001).

Segment 4 cited fear as a barrier most often (77% vs 61%, p<0.001) and were more likely to say that men may not have considered testing (33% vs 26%, p<0.01) or that they would prefer not to know (16% vs 12%, p = 0.02). Segment 4 was most likely to find self-test kits acceptable (blood-based: 71% vs. 66%, p = 0.04; saliva based: 78% vs. 72%, p = 0.005).

The HCD analysis determined that men in Segment 4 have a progressive view of sex, gender and relationships and is optimistic about the future. They know a lot about HIV, but nevertheless do not test for HIV frequently and may not link promptly if they test positive. This may stem from their fear of HIV and the lifestyle they perceive they would have to give up if they were to test positive. Interventions could focus on the idea that HIV does not mean a complete change in lifestyle. Messaging could include the efficacy of HIV medication and U = U (undetectable = untransmittable).

#### Segment 5

Men in Segment 5 were relatively more conservative in their values and outlook, adhering to traditional norms and structures of family and community (belief in traditional masculinity 0.23, p<0.001; belief in use of violence in relationships 0.41, p<0.001; belief that men should rule the household 0.31, p<0.001). This segment was most likely to say that they enjoy risking taking behaviour (0.73, p<0.001).

They had low levels of HIV knowledge (mean score 4.7 vs 6.4, p<0.001) and low testing rates (56.8% in last year vs 64.3%, p<0.001). Their reaction to HIV was dominated by fear, in particular of losing relationships (0.37, p<0.001).

Men in Segment 5 gave several barriers including practical considerations (15% cited distance from clinic vs 11%, p = 0.03; 13% said men may not know where to test vs 9%, p = 0.01) and low risk perception (36% said not feeling at risk of HIV vs. 30%, p = 0.02; 21% said because their partners are not HIV positive vs 14%, p = 0.001). They rejected many modes of testing (testing in the workplace 44% vs 53%, p = 0.003; testing in community gathering places 14% vs 23%, p<0.001; testing at truck stops 16% vs. 25%, p<0.001; testing through sangomas 9% vs 14%, p = 0.03) and did not express a clear preference for any modalities.

HCD analysis found that men in segment 5 approach life traditionally. They are vigilant about HIV risk and report using condoms frequently and testing often, possibly because they are fearful of what an HIV diagnosis could mean for their relationship with their wife or wider community members. Interventions for this segment could emphasise the benefits of knowing one’s status for future health and for the health of one’s family.

## Discussion

This study applied psycho-behavioural segmentation analysis and the principles of Human Centred Design to describe five segments of young men in South Africa, distinct in terms of their attitudes to HIV and their barriers to uptake of HIV testing and treatment. Segment 1 (21%) avoids the topic of HIV, perhaps fearful of the impact on his life. Segment 2 (23%) is well connected to his community and has social concerns about HIV. Segment 3 (15%) struggles with more distal determinants of HIV acquisition such as unemployment and poor mental health. Segment 4 (25%) has concerns about the lifestyle changes that would be required if he were HIV positive. Segment 5 (16%) has a strong traditional mindset and is fearful of the ramifications of HIV in his community.

Consistent with other findings, segments which reported more frequent alcohol consumption, less frequent use of condoms, experiences of depression and higher levels of identification with traditional expressions of masculinity and unfavourable attitudes towards women had a higher proportion of respondents living with HIV [5, 6, 55, 56]. The study also suggests that level of community belonging may be related to HIV testing and treatment behaviour, which to our knowledge has not been comprehensively investigated in the literature. The study also adds a much more granular understanding of how specific beliefs (such as an HIV diagnosis causing disruption to relationships, or concerns about disclosure) may inform HIV-related behaviour, which may be used to address particular concerns through messaging or other interventions.

These data provide initial guidance on how HIV interventions maybe designed for men in South Africa with their needs and preferences in mind, but they should not be operationalised before a more comprehensive design process has been undertaken and thorough piloting among the intended audience has been completed [54]. This is especially true for populations outside of the specific study areas or target populations. Recruitment of men in each segment for testing purposes can be facilitated by the typing tool, which predicts the segment a man falls into from a short battery of questions. This approach has been used to tailor demand creation interventions for VMMC to population subgroups, but the results of a study evaluating its effectiveness has not yet been published [57]. The segmentation results could also be used to consider more distal causes of HIV acquisition (e.g. mental health difficulties, food insecurity); to tailor discussions in existing interventions and routine services by identifying which segment a man falls into; or to inform mass media campaigns by integrating an awareness of the heterogeneity of the target population into the design process.

### Limitations

Despite the contributions of this study to understanding young men who are at risk of HIV in South Africa, this study has several limitations. The results are limited to the eligible men in the two provinces of interest and may not represent the attitudes and behaviours of men outside of these two settings. The study was cross-sectional in design so it is not possible to understand how the distribution or composition of segments may change over time. The sample size of HIV positive men in the survey was small, meaning it is difficult to draw segment-specific conclusions about these men’s experiences.

Several measures in the study were susceptible to recall bias (e.g. frequency of condom use in the last year) or social desirability bias (e.g. number of sexual partners in the last 12 months) which could have had an impact on the final segmentation solution. HIV testing frequency, HIV status and HIV treatment status were self-reported and not externally verified, meaning we are unaware of the extent of misclassification of HIV status by segment, which could alter the interpretation of the results.

The segmentation analysis process produced several segment solutions and the final one was chosen by the study team based on ease of understanding and translation into practical implementation, which introduced bias. As part of the analysis multiple significance tests were conducted, which is likely to have led to Type 1 and Type 2 errors.

Although elements of an established HCD protocol were followed, these methods are not standardised and therefore the process and outputs are open to the bias of the researchers and workshop participants. Although HCD research has been applied in a global health setting, in the absence of evaluative studies there is limited evidence that it leads to better outcomes than interventions designed in more traditional ways.

It should also be noted that the implementation of the study results- through categorising men using the typing tool and delivering targeted messages and interventions based on their segment group- could be challenging logistically and operationally, especially in low resource settings.

## Conclusion

The study contributes to intervention science’s growing focus on tailoring behaviour change campaigns to ensure that they are maximally effective. Further research should be carried out to evaluate the impact of the interventions produced using the segmentation and HCD frameworks and to validate the typing tool. We also recommend that additional research includes external confirmation of HIV status and treatment status (through screening or clinic records) to more rigorously evaluate the relationship between the segments and HIV outcomes. This will provide evidence for implementers and policy-makers to judge the worth of replicating the analysis. As targeted segmentation and HCD-led approaches to developing interventions are in their infancy, research should be carried out in other settings and disease areas to further test the efficacy of these approaches.

## Supporting information

S1 Dataset Full study dataset. (XLSX)

S1 Questionnaire Questionnaire (English). (DOCX)

S1 Table List of segmentation variables. (DOCX)
