# Supplementary material for: Targeting interventions for HIV testing and treatment uptake: An attitudinal and behavioural segmentation of men aged 20–34 in KwaZulu-Natal and Mpumalanga, South Africa
Source: PLoS One. 2021 Mar 10;16(3):e0247483. doi: 10.1371/journal.pone.0247483 (PMC7946194; doi:10.1371/journal.pone.0247483)
Supplement: S1 Questionnaire — (DOCX) [file pone.0247483.s002.docx]

Ipsos Healthcare

3 Thomas More Square, London, E1W 1YW, UK

Tel: + 44 20 3059 5000 Fax: +44 20 3059 4998

Internal client use only

Population Service International

Testing, Treatment and PrEP

Segmentation

18-047385-01

Screener and Questionnaire

KZN & MPU

Quotas- TBC

|  | KwaZulu Natal | Mpumalanga |
| --- | --- | --- |
|  |  |  |
|  |  |  |
| TOTAL |  |  |

60-minute Questionnaire

PRECONTACT

| Item Code | Question | Response options |
| --- | --- | --- |
| ea.code | {autofill from sample database} | Province, district, subdistrict and enumeration area |
| timestamp | {autofill from app} |  |
| HHID | [Enter Household Number] | {numeric box: range 000-999} |
| HH.type | [Enter Type of Dwelling] | [1] Free hold formal house  [2] RDP house  [3] Informal house  [4] Backyard shack  [5] Flats/ Hostels  [6] Traditional rural hut  [9] Other, specify: {text box} |
| CT01 | [Contact Outcome} | [1] Contact made with household member  [2] Contact made with neighbour/ Other  [3] Noncontact |
| CT01R | {If CT01 = 2 or 3}  [ Enter reason for noncontact with Household] | [1] No one home/ answer door  [2] Locked gate/ security guards  [3] Vacant unit  [4] Not safe to enter  [9] Other, specify: { text box } |
| END1 | {if CT01R = 1 or 9}  [Schedule next contact attempt] | [EXIT] |
| END2 | {if CT01R = 2 or 3 or 4}  {Mark contact sheet as REPLACEMENT} | [EXIT] |
| CT02 | { if CT01 = 1 }  Hello, my name is [SAY NAME] and I am working with Ask Afrika, an independent research agency who are working on behalf of an international organization.  We are listening to the views of men aged 20-34 about health issues and would like to interview a man who is between 20-34 years old. Is there a man aged between 20-34 who normally stays in this house? | [1] Yes  [2] No  [-9] Refuse |
|  | {if CT02 not 1, go to END3] |  |
| CT03 | {if CT02= 1]  How many people live in this house in total? | {numeric box: range 1-10}  [-9] Refuse |
| CT04 | {if CT02 = 1}  How many men in this house are between 20-34? | {numeric box: range 1-9, not greater than CT03}  [-9] Refuse |
| CT05 | { if CT04 > 1 }  Of these {CT04.answer} men, who had the most recent birthday in the past 12 months? | [1] Target respondent present  [2] Target respondent away- schedule return visit  [-9] Refuse |
| CT06 | { if CT04 = 1}  Is he here now? | [1] Target respondent present  [2] Target respondent away- schedule return visit  [-9] Refuse |
| CT06R | { if CT05 = 2 or CT6 =2}  [Schedule return visit to interview target respondent] | [1] Save to continue later  [2] Ready to continue now |
|  | { if CT05 = -9 or CT06 = -9, go to END3} |  |
| END 3 | “Thank you for your time.”  {Mark contact sheet as REPLACEMENT} |  |

ON CONTACT

SCREENER

READ SCREENER WITH RESPONDENT AND THEN HAND OVER FOR FINAL CONSENT. MAKE THE RESPONDENT FEEL AT EASE, AND ALSO REITERATE THE IMPORTANCE OF THE RESEARCH.

INTRODUCTION

Who we are

Hello, I am *[interviewer name].* I am working for a research company called Ask Afrika.

What we are doing

We are randomly selecting 2000 men such as yourself, throughout South Africa to conduct a research study attitudes, behaviours and perceptions about health issues. The results of this survey will help make communities like this safer and healthier.

At times, the discussion will cover personal and sensitive topics such as sexual health, sexual behaviours and HIV, but, please, be honest and open when sharing your thoughts and experiences on these topics as it is important for us to understand your real opinions. There are no right or wrong answers.

Your participation

If you agree, we will ask you to participate in an interview for approximately sixty minutes.

Please understand that your participation is voluntary and you are not being forced to take part in this study. The choice of whether to participate or not, is yours alone. If you choose not to take part, you will not be affected in any way whatsoever. If you agree to participate, you may stop participating in the research at any time and tell me that you don’t want to go continue. If you do this, there will be no penalties and you will not be prejudiced in any way.

Confidentiality

In addition to information about your attitudes and behaviour, we will also collect identifying information such as your name, telephone number and address so that we can contact you. You may be recontacted up to a period of 6 months after your participation in the research.

All identifying information will be kept an encrypted server (i.e. on a secure computer) and will not be available to others and will be kept confidential to the extent possible by law. The records from your participation may be reviewed by people responsible for making sure that research is done properly, including members of the ethics committee at FPD. (All of these people are required to keep your identity confidential.) Otherwise, records that identify you will be available only to people working on the study.

We are asking you to give us permission to tape-record the interview so that we can accurately record what is said.

To compensate for your time, participation and any inconvenience you will receive a R60 grocery voucher.

Your deidentified answers will be stored electronically in a secure environment and used for research or academic purposes now or at a later date in ways that will not reveal who you are. All future use of the stored data will be subject to further Research Ethics Committee review and approval.

We will not record your name anywhere and no one will be able to connect you to the answers you give. Your answers will be linked to a fictitious code number or a pseudonym (another name) and we will refer to you in this way in the data, any publication, report or other research output.

Risks/discomforts

The most significant risk is a breach of confidentiality, but we have put measures in place to ensure that this does not happen. We also have plans in the place to limit any negative effects if someone finds out things about you that you do not wish them to know.

We realise that this is sensitive topic, so if you wish to conduct the interview in a different location where people can’t hear you, you may. You may also refuse to answer any of the questions and may stop the interview at any time. We have procedures in place to ensure your wellbeing if you become distressed during the interview.

Benefits

If you would like to receive feedback on our study, we will record your phone number on a separate sheet of paper and can send you the results of the study when it is completed sometime after December 2018. This information will also be stored securely and only viewed by those who are required to see it.

Who to contact if you have been harmed or have any concerns

This research has been approved by the FDP Research Ethics Committee and PSI’s REC. If you have any complaints about ethical aspects of the research or feel that you have been harmed in any way by participating in this study, please call +27 (0) 12 816 9000 or email foundation@foundation.co.za

If you have concerns or questions about the research you may call the project leader Jean Moolman, +27 12 428 7400, Jean.Moolman@askafrika.co.za.

HAND OVER TABLET

| The research has been explained to me and I have been given a full explanation about the possible uses of any personally identifying information | | YES □ NO □  IF “No” then Close |
| --- | --- | --- |
| I consent to participating in this research  {If “No” mark contact sheet as SUBSTITUTE} | | YES □ NO □  IF “No” then Close |
| I consent to the use of my audio recording as described before | | YES □ NO □  IF “No” then Close |
| Are you happy for Ask Afrika to re-contact you regarding this research? | | YES □ NO □ |
| Print full name |  | |
| Signature |  | |
| Date |  | |
| Telephone number | IF PARTICIPANT CONSENTS TO TAKE PART: CREATE RANDOM 6 DIGIT ID CODE | |
| Email address |  | |

THANK YOU. PLEASE HAND SCREEN BACK TO INTERVIEWER

To start, we will ask you a few questions to make sure that this study will be relevant to you.

ASK ALL RESPONDENTS

INTERVIEWER TO CODE

ONLY ONE ANSWER

AUTOMATIC LANGUAGE SWITCH ACCORDING TO LANGUAGE SELECTED

S1. Which language would you prefer to do the interview in?

| English | 1 |
| --- | --- |
| siSwati | 2 |
| Xitsonga | 3 |
| Sepedi | 4 |
| IsiZulu | 5 |

ASK ALL RESPONDENTS

PROGRAMMER TO ADD DROP DOWN OPTIONS WITH MONTH AND YEAR

S2. In which month and year were you born?

| Month | Year |
| --- | --- |

AUTO CALCULATE AGE

PROGR: TERMINATE IF < 20 YEARS OR > 34 YEARS

PROGR.: PLEASE RECODE AGE IN ONE OF THE FOLLOWING INTERVALS

S2.1 AGE

| 20-24 | 1 | CHECK QUOTAS |
| --- | --- | --- |
| 25-29 | 2 | CHECK QUOTAS |
| 30-34 | 3 | CHECK QUOTAS |

ASK ALL RESPONDENTS

SINGLE ANSWER

READ OUT

S3. What is the highest level of schooling you have achieved?

| No formal schooling or qualifications | 1 | CONTINUE |
| --- | --- | --- |
| Some schooling but no matric (did not complete high school) | 2 | CONTINUE |
| Matric (finished high school) | 3 | CONTINUE |
| College/ vocational training (TVET) | 4 | CONTINUE |
| University (Not graduated) | 5 | CONTINUE |
| University (graduated) | 6 | CLOSE |

ASK ALL RESPONDENTS

SINGLE ANSWER

CAPTURE BY OBSERVATION/ ASK IF UNSURE

S4. What is your race?

| Black/ African | 1 | CONTINUE |
| --- | --- | --- |
| Coloured | 2 | CLOSE |
| Indian or Asian | 3 | CLOSE |
| White | 4 | CLOSE |
| Other/ Declined | 5 | CLOSE |

ASK ALL RESPONDENTS

SINGLE ANSWER

CAPTURE SPONTANEOUS ANSWER

S5. The next set of questions can be sensitive to some people, so please do not feel pressured into answering them. We can end the interview at any point.

All answers you enter will be kept confidential and nobody in the community or beyond will find out about the answers you give us to the questions in this survey.

Are you happy to proceed?

| 1 | Yes | CONTINUE |
| --- | --- | --- |
| 2 | No | CLOSE |

ASK IF “No” SELECTED IN S5

CAPTURE OPEN ENDED ANSWER

REF1

| {text box: limit 100 characters} Enter reason for refusal |
| --- |

ASK ALL RESPONDENTS

SINGLE ANSWER

READ OUT OPTIONS

S6. What is your circumcision status?

| 1 | Not circumcised | CONTINUE |
| --- | --- | --- |
| 2 | ~~No~~ Circumcised traditionally | CONTINUE |
| 3 | Circumcised in a clinic before the age of 16 | CONTINUE |
| 4 | Circumcised in a clinic after the age of 16 | CONTINUE |

ASK ALL RESPONDENTS

SINGLE ANSWER

CAPTURE SPONTANEOUS ANSWER

S7. Have you ever heard of the virus HIV or an illness called AIDS?

| 1 | Yes | CONTINUE |
| --- | --- | --- |
| 2 | No | CLOSE |

HAND OVER TABLET

ASK S8c if S8b =1

SINGLE ANSWER

S8c. Have you ever been tested for HIV? Please remember that your answers will be private and please answer honestly, as this will help make the lives of men all over South Africa better

| 1 | Yes | CONTINUE |
| --- | --- | --- |
| 2 | No | CONTINUE TO SECTION A |
| 3 | Prefer not to say | CLOSE |

ASK S9c IF S9b=1

SINGLE ANSWER

S9c. What was the result of your most recent test? Please remember that your answers will be private and please answer honestly, as this will help make the lives of men all over South Africa better

| 1 | Positive | CONTINUE |
| --- | --- | --- |
| 2 | Negative | CONTINUE |
| 3 | Did not receive the results | CONTINUE |

THANK YOU. PLEASE HAND SCREEN BACK TO INTERVIEWER

CREATE VARIABLE FOR HIV STATUS- COMBINE S9A AND S9C

IF QUALIFY THEN TEXT SHOULD READ:-

“Thank you for answering these questions. This study is suitable for you. The survey will take approximately 55 minutes and you will be compensated with a R60 grocery voucher for your time”

At the end of the interview, I will give you an opportunity to keep a self-test kit, if you are HIV negative and you consent to it. This may add an extra 10 minutes at the end.

IF CLOSE THEN TEXT SHOULD READ:-

“Thank you for answering these questions and for your time but unfortunately this survey is not right for you, and we don’t want to take any more of your time. Thank you for your help so far”

SECTION A: Living situation, employment and support network

rEAD OUT TO ALL:

*We’d like to start by understanding a bit more about where you live.*

ASK ALL

SINGLE ANSWER

DO NOT READ LIST

A1. How long have you stayed (lived) in this community?

|  | All his life |
| --- | --- |
|  | More than 15 years |
|  | 10-14 years |
|  | 5-9 years |
|  | 3-4 years |
|  | 1-2 years |
|  | Less than a year |
|  | Prefer not to answer |

ASK if options 4-7 at A1

SINGLE ANSWER

READ LIST- prompt for time it takes to drive rather than distance

A2. Thinking about the place you were born. How far away is that place from here?

|  | In a community more than 2 hours drive away in South Africa |
| --- | --- |
|  | In a community 1-2 hours drive away in South Africa |
|  | In a community less than an hour drive away in South Africa |
|  | In a different country |
|  | Prefer not to answer |

ASK ALL

DISPLAY SHOWCARD ON SCREEN – RATING SCALE

REPEAT SCALE WORDING AS necessary

SHOW RESPONDENT SCREEN

A3. I am going to read you some statements and would like you to tell me how much you disagree or agree with them using a 5 point scale where 5 means strongly agree, 4 means slightly agree, 3 means neither agree nor disagree, 2 means slightly disagree and 1 means strongly disagree:

options across the top

single response per row

|  | Strongly disagree |
| --- | --- |
|  | Slightly disagree |
|  | Neither agree nor disagree |
|  | Slightly agree |
|  | Strongly agree |
|  | Prefer not to answer |

options down the side

randomise list

|  | I like the community where I live |
| --- | --- |
|  | I have friends I trust in the community I live in |
|  | I feel like I do not belong in this community |
|  | I would live somewhere else if I could |
|  | I have an important role to play in this community |

ASK ALL

multiple response

do not read out list, select all which apply

A4. Who do you live with?

|  | Mother |
| --- | --- |
|  | Father |
|  | Grandmother |
|  | Grandfather |
|  | Brother (s) |
|  | Sister (s) |
|  | Wife/ partner/ Husband |
|  | Son(s) |
|  | Daughter(s) |
|  | Other relatives’ children |
|  | Other relative (s) |
|  | Male friend (s) |
|  | Female friend (s) |
|  | Not friend or relative but another man sharing the house |
|  | Not friend or relative but another woman sharing the house |
|  | Other people’s children (not related) |
|  | Live alone [EXCLUSIVE CODE] |
|  | Prefer not to answer |

ASK if option 7 not selected at a4

SINGLE ANSWER

CAPTURE SPONTANEOUS ANSWER

A5. Do you have a wife, husband or partner who you are not currently living with? By partner we mean a main girlfriend/ boyfriend or a mother of your children you consider to be main partner

|  | Yes |
| --- | --- |
|  | No |
|  | Prefer not to answer |

ASK if option 1 selected at a5

SINGLE ANSWER

READ OUT

A6. How far away does your main wife/ husband/ partner live? Please estimate the time it would take by car to get to them

|  | Less than 30 minutes away |
| --- | --- |
|  | 30 minutes to less than 1 hour away |
|  | 1-2 hours away |
|  | More than 2 hours away |
|  | Prefer not to answer |

ASK if options 8 or 9 not selected at a4

SINGLE ANSWER

CAPTURE SPONTANEOUS ANSWER

A7. Do you have children who you are not currently living with?

|  | Yes |
| --- | --- |
|  | No |
|  | Prefer not to answer |

ASK if selected option 8 or 9 at a4 or option 1 at a7

SINGLE NUMERIC RESPONSE PER LINE BETWEEN 0 – 10

AT LEAST ONE RANGE MUST BE >0

CAPTURE NUMERIC ANSWER

A8. How many children do you have within the following age bands? Please include all the children you have whether you live with them or not.

|  | 0-4 |  |
| --- | --- | --- |
|  | 5-10 |  |
|  | 11-14 |  |
|  | 15-18 |  |
|  | 19+ |  |

ASK if options 1 or 2 not selected at a4

SINGLE ANSWER

CAPTURE SPONTANEOUS ANSWER

**A9.2. If option 2 NOT selected:** Is your father still alive?

**A9.3. If option 1 NOT selected:** Is your mother still alive?

| FATHER STILL ALIVE? | |
| --- | --- |
|  | Yes |
|  | No |
|  | Don’t know |
|  | Prefer not to answer |
| Mother STILL ALIVE? | |
|  | Yes |
|  | No |
|  | Don’t know |
|  | Prefer not to answer |

ASK if option 6 selected at a9

numeric response – range 0-current age at s2

CAPTURE NUMERIC ANSWER

A10. How old were you when your father died?

|  | _______ years old |
| --- | --- |
| If respondent can’t remember, see if they can answer with one of the following: | |
|  | A baby/ toddler |
|  | A child |
|  | A teenager |
|  | An adult |
|  | Don’t know |
|  | Prefer not to answer |

ASK if option 10 selected at a9

numeric response – range 0-current age at s2

CAPTURE NUMERIC ANSWER

A11. How old were you when your mother died?

|  | _______ years old |
| --- | --- |
| If respondent can’t remember, see if they can answer with one of the following: | |
|  | A baby/ toddler |
|  | A child |
|  | A teenager |
|  | An adult |
|  | Don’t know |
|  | Prefer not to answer |

ASK all

SINGLE ANSWER

CAPTURE SPONTANEOUS ANSWER

A12. Do you have a steady job (a regular source of income)?

|  | Yes |
| --- | --- |
|  | No |
|  | Prefer not to answer |

ASK if option 1 at A12

SINGLE ANSWER

do not read out list

A13. What industry/ profession do you work in?

|  | Agriculture |
| --- | --- |
|  | Construction |
|  | Mining |
|  | Retail |
|  | Factory production |
|  | Hospitality (catering) |
|  | Security |
|  | Tourism |
|  | Education |
|  | Healthcare |
|  | Taxi |
|  | Government administrator |
|  | Studying |
|  | Traditional leader |
|  | Legal |
|  | Banking/ finance |
|  | Other (SPECIFY) |

ASK if option 2 at A12

numeric response – range 0-30

CAPTURE NUMERIC ANSWER

A13. How many days in the past 30 days did you do any work for pay?

|  | _________ days |
| --- | --- |
|  | Prefer not to answer |

ASK ALL

DISPLAY SHOWCARD ON SCREEN – RATING SCALE

REPEAT SCALE WORDING AS necessary

SHOW RESPONDENT SCREEN

A15. I am going to read you some statements and would like you to tell me how much you disagree or agree with them using a 5 point scale where 5 means strongly agree, 4 means slightly agree, 3 means neither agree nor disagree, 2 means slightly disagree and 1 means strongly disagree:

options across the top

single response per row

|  | Strongly disagree |
| --- | --- |
|  | Slightly disagree |
|  | Neither agree nor disagree |
|  | Slightly agree |
|  | Strongly agree |
|  | Prefer not to answer |
|  | Not Applicable |

options down the side

randomise list

|  | Drinking alcohol helps me belong with the other men in the community |
| --- | --- |
|  | Sometimes I do things I regret when drinking alcohol |
|  | Sometimes I drink so much alcohol I don’t remember what happened |
|  | I spend too much money on alcohol |
|  | Drinking alcohol is bad for my health |
|  | Drinking alcohol makes me forget my worries |
|  | Drinking alcohol makes me feel good |
|  | Drinking alcohol helps me pick up women |
|  | Sometimes when I drink alcohol I feel out of control |

SECTION B: Personality traits, aspiration and behaviours

rEAD OUT TO ALL:

Thank you, now we’d like to understand a little bit more about you as a person. Please be honest and think about how you feel and how others might describe you

Ask ALL

DISPLAY SHOWCARD ON SCREEN – RATING SCALE

REPEAT SCALE WORDING AS necessary

SHOW RESPONDENT SCREEN

B1. I’m going to list several personality traits which may apply or may not apply to you. Please think about how your friends might describe you and how much they would agree or disagree with the following personality traits.

Use a 5 point scale where 5 means strongly agree, 4 means slightly agree, 3 means neither agree nor disagree, 2 means slightly disagree and 1 means strongly disagree:

options across the top

single response per row

|  | Strongly disagree |
| --- | --- |
|  | Slightly disagree |
|  | Neither agree nor disagree |
|  | Slightly agree |
|  | Strongly agree |
|  | Prefer not to answer |

options down the side

Randomise list

|  | Fun, cheerful |
| --- | --- |
|  | Serious, disciplined |
|  | Respected, powerful |
|  | Traditional, caring |
|  | Sociable, approachable |
|  | Stylish, expert |
|  | Adventurous, passionate |
|  | Reliable, trustworthy |

Ask ALL

DISPLAY SHOWCARD ON SCREEN – ANSWER OPTIONS

single response for ‘MOST like you’ and ‘least like you’

OpTION FOR ‘MOST like you’ CANNOT BE SELECTED FOR ‘least like you’

SHOW RESPONDENT SCREEN

B2. From this list, which is the MOST like you and which is the LEAST like you?

|  | Fun, cheerful |
| --- | --- |
|  | Serious, disciplined |
|  | Respected, powerful |
|  | Traditional, caring |
|  | Sociable, approachable |
|  | Stylish, expert |
|  | Adventurous, passionate |
|  | Reliable, trustworthy |

Ask ALL

DISPLAY SHOWCARD ON SCREEN – RATING SCALE

ADD B3 STATEMENTS 1 AND 2 TO GIVE ‘ENJOYMENT SCORE’, 3 AND 4 TO GIVE ‘CONTROL SCORE’, 5 AND 6 TO GIVE ‘POWER SCORE’, 7 AND 8 TO GIVE ‘BELONGING SCORE’, 9 AND 10 TO GIVE ‘CONVIVIALITY SCORE’, 11 AND 12 TO GIVE ‘RECOGNITION SCORE’, 13 AND 14 TO GIVE ‘VITALITY SCORE’, 15 AND 16 TO GIVE ‘SECURITY SCORE’. ADD 5 FOR MOST LIKE YOU AND TAKE 5 FOR LEAST LIKE YOU

REPEAT SCALE WORDING AS necessary

SHOW RESPONDENT SCREEN

B3. I am now going to list several statements about different outlooks on life. Please think about how much you agree or disagree with the following statements and how closely they reflect your outlook on life.

Use a 5 point scale where 5 means strongly agree, 4 means slightly agree, 3 means neither agree nor disagree, 2 means slightly disagree and 1 means strongly disagree:

options across the top

single response per row

|  | Strongly disagree |
| --- | --- |
|  | Slightly disagree |
|  | Neither agree nor disagree |
|  | Slightly agree |
|  | Strongly agree |
|  | Prefer not to answer |

options down the side

Randomise list

|  | I like to have as much fun as possible in life |
| --- | --- |
|  | I like to take every day as it comes |
|  | I like to have lots of order and structure in my life |
|  | I like to collect lots of facts before making a decision |
|  | People consider me a leader rather than a follower |
|  | I like to stand out from the rest |
|  | Being an active member of the local community is important to me |
|  | It is important to honour and continue my family traditions |
|  | I enjoy meeting new people |
|  | Having good friends is more important that having lots of money |
|  | It is more important to be unique than to be popular |
|  | I am not afraid to be different to others |
|  | I am constantly looking for new experiences |
|  | I believe that the world is full of exciting opportunities |
|  | I enjoy spending time by myself to relax |
|  | I like a steady and stable life |

ASK ALL

DISPLAY SHOWCARD ON SCREEN – ANSWER OPTIONS

multiple response FOR TWO RESPONSES

RANDOMISE

SHOW RESPONDENT SCREEN

SELECT 2 ONLY

B4. Which two of these statements best describes your outlook on life?

|  | I like to have as much fun as possible in life |
| --- | --- |
|  | I like to take every day as it comes |
|  | I like to have lots of order and structure in my life |
|  | I like to collect lots of facts before making a decision |
|  | People consider me a leader rather than a follower |
|  | I like to stand out from the rest |
|  | Being an active member of the local community is important to me |
|  | It is important to honour and continue my family traditions |
|  | I enjoy meeting new people |
|  | Having good friends is more important that having lots of money |
|  | It is more important to be unique than to be popular |
|  | I am not afraid to be different to others |
|  | I am constantly looking for new experiences |
|  | I believe that the world is full of exciting opportunities |
|  | I enjoy spending time by myself to relax |
|  | I like a steady and stable life |

Ask ALL

DISPLAY SHOWCARD ON SCREEN – RATING SCALE

REPEAT SCALE WORDING AS necessary

SHOW RESPONDENT SCREEN

B5. I’m now going to read out some statements. For each, please let me know the extent you agree or disagree.

Use a 5 point scale where 5 means strongly agree, 4 means slightly agree, 3 means neither agree nor disagree, 2 means slightly disagree and 1 means strongly disagree:

options across the top

single response per row

|  | Strongly disagree |
| --- | --- |
|  | Slightly disagree |
|  | Neither agree nor disagree |
|  | Slightly agree |
|  | Strongly agree |
|  | Prefer not to answer |

options down the side

Randomise list

|  | I get enjoyment from taking risks |
| --- | --- |
|  | When I am upset I can act without thinking |
|  | I do what my family expects of me |
|  | Success is due to hard work, not luck |
|  | I am not sure what I want to do with my life |
|  | When something scares me, I avoid thinking about it |
|  | I will do what is good for my community even if it may not benefit me |
|  | In 5 years time, I will be better off than I am now |
|  | I am proud of my achievements so far |
|  | I have goals I want to achieve in my career |
|  | I want to live to see my children grow up or have children in the future |

Ask ALL

DISPLAY SHOWCARD ON SCREEN – RATING SCALE

REPEAT SCALE WORDING AS necessary

SHOW RESPONDENT SCREEN

B6. When thinking about relationships between men and women, to what extent do you agree or disagree with the following statements?

Use a 3 point scale where 1 means somewhat agree, 2 means strongly agree and 3 means do not agree

options across the top

single response per row

|  | Somewhat agree |
| --- | --- |
|  | Strongly agree |
|  | Do not agree |

options down the side

randomise list

|  | Woman’s most important role is to take care of her home and cook |
| --- | --- |
|  | Men need sex more than women do |
|  | Men don’t talk about sex, they just do it |
|  | There are times when a woman deserves to be beaten |
|  | Changing nappies, giving kids a bath and feeding kids are the mother’s responsibility |
|  | It is a woman’s responsibility to avoid getting pregnant |
|  | A man should have the final word about decisions in his home |
|  | Men are always ready to have sex |
|  | A woman should tolerate violence in order to keep her family together |
|  | I would be outraged if my wife asked me to use a condom |
|  | A man and a woman should decide together what contraceptive to use |
|  | I would never have a gay friend |
|  | If someone insults me, I will defend my reputation, with force if I need to |
|  | To be a man, you need to be tough |
|  | Men should be embarrassed if unable to get an erection |
|  | If a guy gets a woman pregnant, the child is the responsibility of both |
|  | Man should know what his partner likes during sex |
|  | Woman should know what her partner likes during sex |
|  | The participation of the father is important in raising children |
|  | It’s important for man to have friends to talk about his problems |
|  | Couples should decide together if they want to have children |

ASK ALL

ENTER 999 IF DON’T KNOW OR NO ANSWER. ENSURE AGE DOES NOT EXCEED AGE AT S2

CAPTURE NUMERIC ANSWER

ENTER 999 IF DON’T KNOW OR NO ANSWER

B7. I now want to ask some sensitive questions about having sex. To the best of your memory, how old were you when you first had sex?

| _______ | years old |
| --- | --- |

ASK ALL

numeric answer – range 0-300

CAPTURE NUMERIC ANSWER

IF RESPONDENT GIVES A RANGE, TAKE THE MID POINT. E.G. IF RESPONDENT SAYS 10-15, GO FOR 12 OR 13

B8. How many sexual partners have you had in the past 12 months?

|  | ______ sexual partners in past 12 months |
| --- | --- |

ASK if b8 >0

numeric answer; must be less than answer at b9

CAPTURE NUMERIC ANSWER

B9. How many people are you currently having regular sex with? By this, we mean that you have had sex with them before and you will likely have sex with them again in the future. Do not include people you think you will only have sex with once.

|  | ______ current sexual partners |
| --- | --- |

NEW SCREEN

READ OUT: Different people have different sexual preferences, and this is ok. Some men in this community may like to have sex only with women, some only with men, and others with both women and men.

The next questions are sensitive, and I will hand you the device. You will be asked to describe your most recent sexual partners, up to a maximum of 5, including whether they were female or a male. Please include those you are having regular sex with and also those one-off encounters

If you prefer, I can read the questions to you and input your answers otherwise I will not be able to see your answer if you would rather answer yourself.

HAND OVER TABLET

For each of your sexual partners, can you describe your relationship with them…

ASK if b8 >0.

Ask b10-b14 for up to the last 5 partners maximum

SINGLE ANSWER

B10. What gender are they?

|  | Female |
| --- | --- |
|  | Male |
|  | Another |
|  | Prefer not to answer |

ASK if b8 >0.

Ask b10-b14 for up to the last 5 partners maximum

SINGLE ANSWER

B11. What relation are they to you?

|  | Wife/ husband/ main partner |
| --- | --- |
|  | Another regular girlfriend/ boyfriend |
|  | Casual hookup/ one night stand |
|  | Sex worker |
|  | Blessee / slay queen / makhwepeni |
|  | Blesser |
|  | Any other answer you can think of? |
|  | Prefer not to answer |

ASK if b8 >0.

Ask b10-b14 for up to the last 5 partners maximum

numeric answer – have following options repeated for each partner

B12. How old are they. If you don’t know then please guess?

|  | ______ years old |
| --- | --- |
|  | I don’t know |
|  | Prefer not to answer |

ASK if b8 >0.

Ask b10-b14 for up to the last 5 partners maximum

Grid question – 1 column per partner from B9 across top with partners distinguished by age entered at B10

randomise list

multiple response

B13. Thinking about them, have you ever given them, as a gift, any of the following?

|  | Alcoholic drinks |
| --- | --- |
|  | Cigarettes |
|  | Chocolates |
|  | Fast food |
|  | Soft drugs (e.g. Dagga) |
|  | Hard drugs (e.g. nyaope, crystal meth, heroin, cocaine) |
|  | Clothes, shoes or fashion accessories |
|  | Hair weaves |
|  | Airtime |
|  | Phone |
|  | Money |
|  | Groceries |
|  | Meals in restaurants |
|  | Cosmetics or perfumes |
|  | Holiday trips |
|  | Any other answer you can think of? ANCHOR AT BOTTOM OF LIST |
|  | None of the above ANCHOR AT BOTTOM OF LIST |
|  | Prefer not to answer ANCHOR AT BOTTOM OF LIST |

ASK if b8 >0.

Ask b10-b14 for up to the last 5 partners maximum

SINGLE ANSWER

B14a. How regularly, if at all, did you wear a condom with them?

|  | Never/ didn’t |
| --- | --- |
|  | Once or twice |
|  | Some of the time |
|  | Regularly but not always |
|  | All of the time |
|  | Don’t know/ Prefer not to answer |

ASK IF CODED 1,2,3 OR 4 at B14a

multiple response

randomise responses

B14b. For which reasons did you not wear a condom with them every time? Select all that apply

|  | I was under the influence of alcohol |
| --- | --- |
|  | They asked me not to wear one |
|  | I don’t like condoms |
|  | We didn’t have any condoms |
|  | They looked healthy |
|  | They are faithful to me |
|  | I was under the influence of drugs |
|  | We forgot in the moment |
|  | I trust them |
|  | We were trying to have a baby |
|  | They mentioned they were on the Pill |
|  | Any other answer you can think of? ANCHOR AT BOTTOM OF LIST |
|  | Prefer not to answer ANCHOR AT BOTTOM OF LIST |

THANK YOU. PLEASE HAND SCREEN BACK TO INTERVIEWER

Ask ALL

DISPLAY SHOWCARD ON SCREEN – RATING SCALE

REPEAT SCALE WORDING AS necessary

SHOW RESPONDENT SCREEN

B15. How easy do you find it to talk about sexual health with each of the following people? For example, using condoms, protecting from HIV, protecting from other sexually transmitted infections etc.

options across the top

single response per row

|  | Very difficult |
| --- | --- |
|  | Somewhat difficult |
|  | Neither easy nor difficult |
|  | Fairly easy |
|  | Very easy |
|  | Prefer not to answer |
|  | Not relevant to respondent |

Options down the side

randomise list

|  | Your main sexual partner/ wife |
| --- | --- |
|  | Other regular sexual partners |
|  | Casual sexual partners |
|  | Your male friends |
|  | Your female friends |
|  | Older male family members/ relatives |
|  | Older female family members/ relatives |
|  | Younger male family members/ relatives |
|  | Younger female family members/ relatives |
|  | A healthcare worker or nurse |
|  | A traditional healer |

Ask ALL

DISPLAY SHOWCARD ON SCREEN – RATING SCALE

REPEAT SCALE WORDING AS necessary

SHOW RESPONDENT SCREEN

B16. In an average week, how regularly do you do the following activities?

Options across the top

single response per row

|  | Never (0 days) |
| --- | --- |
|  | Every 1-3 days |
|  | Every 4-6 days |
|  | Every day |
|  | Prefer not to answer |

Options down the side

randomise list

|  | Play soccer |
| --- | --- |
|  | Play snooker/ pool |
|  | Watch TV |
|  | Spend time with my children [ONLY ASK IF 1 CHILD OR MORE AT A8] |
|  | Spend time with my wife/ partner [ONLY ASK IF CODE 1 or 3 selected at A5] |
|  | Drink alcohol with my friends |
|  | Smoke Dagga |
|  | Drink alcohol alone |
|  | Spend time at home alone |
|  | Go to church |
|  | Pray by myself |
|  | Helping other people |
|  | Work |

Ask ALL

DISPLAY SHOWCARD ON SCREEN – RATING SCALE

- ADD ‘PESSIMISM VARIABLE’ BY ADDING SCORES FROM SCALE FROM 3,7 AND 9
- ADD ‘OPTIMISM VARIABLE’ BY ADDING SCORES FROM SCALE 1,4 AND 10

REPEAT SCALE WORDING AS necessary

SHOW RESPONDENT SCREEN

B17. Please indicate to what extend you agree or disagree with the following. Please be as honest and accurate as you can throughout. Try not to let your response to one

statement influence your responses to other statements. There are no "correct" or "incorrect"

answers. Answer according to your own feelings, rather than how you think "most people"

would answer.

Options across the top

single response per row

|  | I disagree a lot |
| --- | --- |
|  | I disagree a little |
|  | I neither agree nor disagree |
|  | I agree a little |
|  | I agree a lot |

Options down the side

randomise list

|  | In uncertain times, I usually hope for the best |
| --- | --- |
|  | It's easy for me to relax |
|  | Lots of bad things seem to happen in my life |
|  | I'm always optimistic about my future |
|  | I enjoy my friends a lot |
|  | It's important for me to keep busy |
|  | I hardly ever expect things to go my way |
|  | I don't get upset too easily |
|  | I rarely count on good things happening to me |
|  | Overall, I expect more good things to happen to me than bad |

SECTION C: Health and wellbeing & Attitudes and knowledge towards HIV

ask all

SINGLE ANSWER

read out

C1. Generally, how would you describe your state of health?

|  | Very healthy and rarely get sick |
| --- | --- |
|  | Generally healthy but occasionally get sick |
|  | I get sick quite often but when I’m not sick I am in good health |
|  | I get sick quite often and even when I’m not sick I am in poor health |
|  | Generally in poor health |
|  | Very sick |
|  | Prefer not to answer |

Ask ALL

DISPLAY SHOWCARD ON SCREEN – RATING SCALE

TOTAL SCORE FROM EACH OPTION USING 0-3 SCALE:

- SCORE OF 0-4= MINIMAL TO NO DEPRESSION, SCORE OF 5-9 = MILD DEPRESSION, SCORE OF 10-14 = MODERATE DEPRESSION, SCORE OF 15-19 = MODERATELY SEVERE DEPRESSION, SCORE OF 20-27 = SEVERE DEPRESSION

REPEAT SCALE WORDING AS necessary

SHOW RESPONDENT SCREEN

C2. Over the last 2 weeks, how often have you experienced any of the following problems?

Options across the top

single response per row

|  | Not at all/ rarely (1 day) |
| --- | --- |
|  | Several days (2-6 days) |
|  | More than half the days (7-11 days) |
|  | Nearly every day (12-14 days) |

Options down the side

RANDOMISE

|  | Little interest or pleasure in doing things |
| --- | --- |
|  | Feeling down, depressed or hopeless |
|  | Trouble falling or staying asleep, or sleeping too much |
|  | Feeling tired or having little energy |
|  | Poor appetite or overeating |
|  | Feeling bad about yourself — or that you are a failure or have let yourself or your family down |
|  | Trouble concentrating on things, such as holding a conversation or completing a task |
|  | Moving or speaking more slowly. Or the opposite — being so fidgety or restless that you move around a lot more than usual |
|  | Thoughts that you would be better off dead or of hurting yourself in some way |

ask all

SINGLE ANSWER

CAPTURE SPONTANEOUS ANSWER

C3. Have you visited a Sangoma in the last 12 months?

|  | Yes |
| --- | --- |
|  | No |
|  | Prefer not to answer |

ask all

SINGLE ANSWER

CAPTURE SPONTANEOUS ANSWER

C4. Have you visited a health clinic in the last 12 months?

|  | Yes |
| --- | --- |
|  | No |
|  | Prefer not to answer |

ask all

SINGLE ANSWER

CAPTURE SPONTANEOUS ANSWER

C5. How frequently, if at all, do you visit a healthcare clinic?

|  | Never been to a clinic |
| --- | --- |
|  | I’ve been once or twice ever |
|  | Once a year |
|  | 2 or 3 times a year |
|  | More than 3 times a year |
|  | Prefer not to answer |

ask if not option 1 at c5

SINGLE ANSWER

CAPTURE SPONTANEOUS ANSWER

C6. When you visit a healthcare clinic, do you always visit the closest clinic or will you sometimes travel further to visit a different clinic?

|  | I always visit my closest clinic |
| --- | --- |
|  | I usually visit my closest clinic but have sometime visited one further away |
|  | I usually travel to visit one further away |
|  | I never go to my closest clinic and always travel to one further away |
|  | Prefer not to answer |

ask if not option 1 at c5 AND NOT option 1 at c6

multiple response

Do not read out list

C7. Why would you visit a clinic a bit further away rather than go to your closest one?

|  | They have better facilities |
| --- | --- |
|  | You don’t have to wait so long |
|  | I prefer the nurses/ doctors there |
|  | So no one sees me going there |
|  | So no one there knows me |
|  | They are more discrete there |
|  | I don’t trust the people at my local clinic not to talk about me |
|  | It is closer to where I work |
|  | It is close to where I used to live |
|  | Other 1 – specify |
|  | Other 2 – specify |
|  | Other 3 – specify |
|  | Prefer not to answer |

ask if not option 1 at c5

SINGLE ANSWER

CAPTURE SPONTANEOUS ANSWER

C8. How long does it take for you to get to the clinic you visit most often? Assume this is by car.

|  | Less than 15 minutes |
| --- | --- |
|  | Between 15 mins to half an hour |
|  | Between half an hour and an hour |
|  | Between 1 and 2 hours |
|  | More than 2 hours |

ASK ALL

DISPLAY SHOWCARD ON SCREEN – RATING SCALE

REPEAT SCALE WORDING AS necessary

SHOW RESPONDENT SCREEN

C9. How much do you agree or disagree with the following statements? 1 means strongly disagree, 2 means disagree, 3 means neither disagree nor agree, 4 means agree, 5 means strongly agree

Options across the top

single response per row

|  | Strongly disagree |
| --- | --- |
|  | Disagree |
|  | Neither agree nor disagree |
|  | Agree |
|  | Strongly agree |
|  | Not applicable |

Options down the side

randomise list

|  | I would feel comfortable with my sangoma giving me a HIV test |
| --- | --- |
|  | I would feel fine with my sangoma recommending me to take western medicine |
|  | A healthcare provider has recommended I see a sangoma before |
|  | A sangoma has recommended I see a healthcare provider before |
|  | I have been treated poorly by a sangoma |
|  | I have been treated poorly by a nurse or other healthcare provider |
|  | My sangoma can see everything about me |
|  | It doesn’t matter if the healthcare provider is an older woman |
|  | It doesn’t matter if the healthcare provider is a man or a womn |

read out to all:

*Let’s now focus on HIV. We’d like to understand more about your views on the topic and not to judge you so please speak as openly as you can.*

ask all

- IF SELECT 1 FOR ATTRIBUTES 2, 4, 5, 11, 12 THEN PLUS 1 POINT. IF SELECT 2 FOR ATTRIBUTES 2, 4, 5, 11, 12 THEN MINUS 1 POINT.
- IF SELECT 1 FOR ATTRIBUTES 1, 3, 6, 7, 8, 9, 10, 13, 14 THEN MINUS 1 POINT. IF SELECT 2 FOR ATTRIBUTES 1, 3, 6, 7, 8, 9, 10, 13, 14 THEN PLUS 1 POINT.
- IF SELECT 3 OR 4 FOR ANY ATTRIBUTES, SCORE 0 FOR THAT ATTRIBUTE.
- TOTAL THE PLUSES AND MINUSES TO GIVE THE RESPONDENT 1 TOTAL SCORE.

DISPLAY SHOWCARD ON SCREEN – RATING SCALE

REPEAT SCALE WORDING AS necessary

SHOW RESPONDENT SCREEN

C10. I’m going to start with a series of statements about HIV and I’d like you to tell me if you think they are true or false.

options across the top

single response per row

|  | True |
| --- | --- |
|  | False |
|  | Don’t Know |
|  | Prefer not to answer |

options down the side

randomise list

|  | HIV can be spread by biting insects, such as mosquitoes or bedbugs. |
| --- | --- |
|  | HIV can be spread by sharing needles or syringes. |
|  | HIV can be spread by sharing food or eating utensils. |
|  | HIV can be spread to babies if they take breast milk of infected mothers. |
|  | Having another STI makes a person more at risk of getting HIV. |
|  | People who have a certain blood type cannot get HIV. |
|  | HIV tests can detect the HIV virus within 24 hours after sex. |
|  | Everyone with HIV will eventually die of AIDS. |
|  | Muthi can protect against HIV. |
|  | Anal sex has a lower risk of spreading HIV than vaginal sex. |
|  | People who have HIV can live to old age if they take medication every day |
|  | People living with HIV and taking treatment are much less likely to pass on HIV to their sexual partners than people with HIV who don’t take treatment. |
|  | Eating certain foods increases/reduces the risk of contracting HIV |
|  | Having sex with certain people can cure HIV |

ASK ALL

DISPLAY SHOWCARD ON SCREEN – RATING SCALE

REPEAT SCALE WORDING AS necessary

SHOW RESPONDENT SCREEN

C11. I’m going to read several statements about HIV. After I read each of them, please tell me how much you agree or disagree with each of them by rating your level of agreement with the statement. Use a 5-point scale, where 1 means ‘Strongly Disagree’, 2 means ‘disagree, 3 means ‘Neither Agree Nor Disagree, 4 means agree and 5 means ‘Strongly Agree’. (Select one)

Options across the top

single response per row

|  | Strongly disagree |
| --- | --- |
|  | Disagree |
|  | Neither agree nor disagree |
|  | Agree |
|  | Strongly agree |

Options down the side

randomise list

| 1 | HIV is a big problem in our community |
| --- | --- |
| 3 | The fear of HIV makes me feel nervous about engaging in sex |
| 4 | The risk of getting HIV hasn’t really affected my behavior |
| 5 | It is difficult to control whether you get HIV or not - even if I do my best I still can get it |
| 6 | It is our duty towards society to prevent HIV |
| 7 | Even if a person has HIV, it is fine for them to wait until they feel ill before going on treatment |
| 8 | Men with HIV find it difficult to find long term partners or settle down |
| 9 | Having HIV often leads to the person being rejected by their community |
| 10 | Once you have an HIV diagnosis, it is impossible to keep it secret, even if you want to |
| 11 | Having HIV means you have to completely change the way you live your life |
| 12 | The impact HIV has on a person’s life is worse than the impact it has on their health |
| 13 | If it is not making you sick, it is better not to know your HIV status than to have to deal with a positive diagnosis |
| 14 | People who get HIV have only got themselves to blame |
| 15 | I would think differently about one of my friends if I knew they had HIV |

ask all

numeric answer – range 0-99 per row

capture numeric value

C12. For the next few questions we’d like you to just give us your best guess at the answer. No one knows the answer to these questions for sure so there is no right or wrong answer. Just tell us what you think. At what age do you think most men and most women get HIV?

|  | MEN: _______ years |
| --- | --- |
|  | WOMEN: _______ years |
|  | Prefer not to answer |

ask all

numeric answer – range 0-100

capture numeric value

C13a. Out of 100 men within your age group, how many do you think have HIV in your local community?

| _______ men |
| --- |
| Prefer not to answer |

ask all

numeric answer – range 0-100

capture numeric value

C13b. Out of 100 women within your age group, how many do you think have HIV in your local community?

| _______ women |
| --- |
| Prefer not to answer |

ask if not HIV positive IN S9a or s9c

SINGLE ANSWER

READ OUT

C14. How likely do you think you are to contract HIV in the next 10 years?

|  | Not at all likely |
| --- | --- |
|  | Unlikely |
|  | Somewhat likely |
|  | Likely |
|  | Very likely |
|  | I have no idea |
|  | Prefer not to answer |

ask all

SINGLE ANSWER

CAPTURE SPONTANEOUS ANSWER

C15. Among your family or close friends, has anyone died of AIDS?

|  | Yes |
| --- | --- |
|  | No |
|  | I suspect they died of HIV/AIDS but don’t know for sure |
|  | Prefer not to answer |

SECTION D: Testing History and attitudes towards testing (tested before)

ASK TO MEN WHO HAVE PREVIOUSLY TESTED FOR HIV ONLY (1 at S8a or 1 at S8c)

READ OUT: In this next section I specifically want to talk about HIV testing.

ROTATE ORDER OF D1 AND D2

ASK TO MEN WHO HAVE PREVIOUSLY TESTED FOR HIV ONLY (1 at S8a or 1 at S8c)

CAPTURE OPEN ENDED ANSWER IN ENGLISH

PLEASE PROBE

D1a. What, if anything, BAD have you heard about visiting clinics for HIV testing?

What else?

|  |
| --- |

ASK TO MEN WHO HAVE PREVIOUSLY TESTED FOR HIV ONLY (1 at S8a or 1 at S8c)

CAPTURE OPEN ENDED ANSWER IN ENGLISH

PLEASE PROBE

D1b. What, if anything, BAD have you heard about HIV testing outside of clinics?

What else?

|  |
| --- |

ASK TO MEN WHO HAVE PREVIOUSLY TESTED FOR HIV ONLY (1 at S8a or 1 at S8c)

CAPTURE OPEN ENDED ANSWER IN ENGLISH

PLEASE PROBE

D2a. What, if anything, GOOD have you heard about visiting clinics for HIV testing?

What else?

|  |
| --- |

ASK TO MEN WHO HAVE PREVIOUSLY TESTED FOR HIV ONLY (1 at S8a or 1 at S8c)

CAPTURE OPEN ENDED ANSWER IN ENGLISH

PLEASE PROBE

D2b. What, if anything, GOOD have you heard about HIV testing outside of clinics?

What else?

|  |
| --- |

ASK TO MEN WHO HAVE PREVIOUSLY TESTED FOR HIV ONLY (1 at S8a or 1 at S8c)

NUMERIC VALUES 1-999

capture numeric value

D3a. How many times have you ever tested for HIV in your life?

| 1 |  |
| --- | --- |

ASK TO MEN WHO HAVE PREVIOUSLY TESTED FOR HIV ONLY (1 at S8a or 1 at S8c)

UP TO 2 ANSWERS ALLOWED EXCEPT CODE 9 AND CODE 10 WHICH ARE MUTUALLY EXCLUSIVE

DISPLAY SHOWCARD ON SCREEN – oPTION LIST

RANDOMISE OPTION

SHOW RESPONDENT SCREEN

D4. What, if anything, is the benefit to having a HIV test? Please select up to your top 2 options from the list below

|  | Knowing HIV status helps me be safe |
| --- | --- |
|  | Knowing HIV status helps me control my health |
|  | Testing for HIV is the smart thing to do |
|  | Knowing my status means that I can be successful |
|  | Knowing my status means I can continue to enjoy life without getting sick |
|  | Knowing my status helps me enjoy life without worry |
|  | Knowing my status builds trust with my partner |
|  | Testing for my status helps me keep my family happy |
|  | Don’t know |
|  | There are no benefits |

ASK TO MEN (WHO HAVE PREVIOUSLY TESTED FOR HIV (1 at S8a or 1 at S8c)) AND (ARE HIV NEGATIVE/ DID NOT RECEIVE THE RESULTS (CODED 2/3 AT S9A OR 2/3 AT S9C))

DISPLAY SHOWCARD ON SCREEN – RATING SCALE

REPEAT SCALE WORDING AS necessary

SHOW RESPONDENT SCREEN

D5a. I am going to read you some statements about HIV testing and would like you to tell me how much you disagree or agree with them. Please use a 5 point scale where 5 means strongly agree, 4 means agree, 3 means neither agree nor disagree, 2 means disagree and 1 means strongly disagree:

options across the top

single response per row

|  | Strongly disagree |
| --- | --- |
|  | Disagree |
|  | Neither agree nor disagree |
|  | Agree |
|  | Strongly agree |
|  | Prefer not to answer |

options down the side

randomise list

|  | Testing for HIV is a very difficult experience |
| --- | --- |
|  | I know who to turn to if I test positive for HIV |
|  | I understand the language healthcare providers use about HIV |
|  | I would feel more comfortable testing with my friends rather than on my own |
|  | I would feel more comfortable testing with my partner rather than on my own |
|  | I would rather test away from where I stay |
|  | If all my friends were going to test, then I would test too |
|  | I wouldn’t want to test incase I was HIV positive |
|  | I am worried that I will no longer be me if I find out I am HIV positive |
|  | I am worried I will die if I find out I am HIV positive |
|  | It is better to know if I am HIV positive than not to know |
|  | I would test if my partner wanted me to |
|  | I would test if the clinics were open at more convenient times |
|  | I would test if I could guarantee nobody would see me at the clinic |
|  | I do not have the time to test for HIV |
|  | I know I should test but often forget |
|  | I would test if I knew that my results would be kept private. |
|  | I would test at a clinic specifically for men |
|  | I would test at a tavern |
|  | I would test in a mall |
|  | I would test at a taxi rank |
|  | After testing negative, I feel my risk is less |
|  | I do not like to test as I do not like needles |

ASK TO MEN WHO HAVE PREVIOUSLY TESTED FOR HIV ONLY (1 at S8a or 1 at S8c)

ask hiv+ respondents only (coded 1 at s9a, or 1 at s9c)

DISPLAY SHOWCARD ON SCREEN – RATING SCALE

REPEAT SCALE WORDING AS necessary

SHOW RESPONDENT SCREEN

D5B. I am going to read you some statements about HIV testing and would like you to tell me how much you disagree or agree with them. Please answer from how you felt before you found out you were HIV Positive. Please use a 5 point scale where 5 means strongly agree, 4 means agree, 3 means neither agree nor disagree, 2 means disagree and 1 means strongly disagree:

options across the top

single response per row

|  | Strongly disagree |
| --- | --- |
|  | Disagree |
|  | Neither agree nor disagree |
|  | Agree |
|  | Strongly agree |
|  | Prefer not to answer |

options down the side

randomise list

|  | Testing for HIV is a very difficult experience |
| --- | --- |
|  | I knew who to turn to if I tested positive for HIV |
|  | I understood the language healthcare provider use about HIV |
|  | I felt more comfortable testing with my friends rather than on my own |
|  | I felt more comfortable testing with my partner rather than on my own |
|  | I would rather test away from where I stay |
|  | If all my friends were going to test, then I would test too |
|  | I didn’t want to test incase I was HIV positive |
|  | I was worried that I will no longer be me if I find out I am HIV positive |
|  | I was worried I will die if I find out I am HIV positive |
|  | It is better to know if I am HIV positive than not to know |
|  | I would test if my partner wanted me to |
|  | I would test if the clinics were open at more convenient times |
|  | I would test if I could guarantee nobody would see me at the clinic |
|  | I did not have the time to test for HIV |
|  | I knew I should test but often forget |
|  | I would test if I knew that my results would be kept private. |
|  | I would test at a clinic specifically for men |
|  | I would test at a tavern |
|  | I would test in a mall |
|  | I would test at a taxi rank |
|  | I wouldn’t test if they use a needle |
|  | After testing I felt that my risk was less |

ASK TO MEN WHO HAVE PREVIOUSLY TESTED FOR HIV ONLY (1 at S8a or 1 at S8c)

SINGLE ANSWER

CAPTURE SPONTANEOUS ANSWER

D6. Have you ever felt pressured into taking a HIV test when you didn’t want to?

|  | Yes |
| --- | --- |
|  | No |
|  | Prefer not to answer |

ASK TO MEN WHO HAVE PREVIOUSLY TESTED FOR HIV ONLY (1 at S8a or 1 at S8c)

SINGLE ANSWER

CAPTURE SPONTANEOUS ANSWER

D7. How long ago was your most recent test?

|  | Within the last 30 days |
| --- | --- |
|  | Within the last 2-3 months |
|  | Within the last 4-6 months |
|  | Within the last 7-12 months |
|  | Within the last 1-2 years |
|  | Within the last 2-3 years |
|  | More than 3 years ago |
|  | Can’t remember |
|  | Prefer not to answer |

ASK TO MEN WHO HAVE PREVIOUSLY TESTED FOR HIV ONLY (1 at S8a or 1 at S8c)

multiple response

Select all which apply

Do not read out list

D8a. Why did you go for your most recent HIV test?

|  | Just a regular, routine test |
| --- | --- |
|  | I had sex without a condom |
|  | I was not feeling well |
|  | I found out a recent sexual partner had HIV |
|  | I found out that my sexual partner had other partners |
|  | My partner insisted I had a test |
|  | I tested with my partner |
|  | My friends went for a test |
|  | Required for an insurance or medical aid or funeral policy |
|  | A healthcare worker in my community advised me to |
|  | It was required for VMMC (VOLUNTARY CIRCUMCISION IN HEALTH CLINIC) |
|  | Mother encouraged me to test |
|  | Father encouraged me to test |
|  | Sister encouraged me to test |
|  | Brother encouraged me to test |
|  | Grandmother encouraged me to test |
|  | Grandfather encouraged me to test |
|  | I was tested without me agreeing |
|  | I went to the clinic for another reason and was offered to test |
|  | Because healthcare workers set up testing locations in places where I was that day |
|  | Other 1 – specify |
|  | Other 2 – specify |
|  | Other 3 – specify |
|  | Can’t remember |
|  | Prefer not to answer |

ASK TO MEN WHO HAVE PREVIOUSLY TESTED FOR HIV ONLY (1 at S8a or 1 at S8c)

SINGLE RESPONSE

Do not read out list

D8b. Where did you do the test?

|  | In a healthcare facility |
| --- | --- |
|  | At work |
|  | In a tent set up by healthcare workers |
|  | At home |
|  | Other [SPECIFY] |

ASK TO MEN WHO HAVE PREVIOUSLY TESTED FOR HIV ONLY (1 at S8a or 1 at S8c)

multiple response

Select all which apply / PROBE FOR AS MANY RESPONSES AS POSSIBLE

Do not read out list

D9. Aside from wanting to know their HIV status, what other reasons are there why men go for HIV tests?

|  | Some go as routine |
| --- | --- |
|  | Their sex partner looked unwell |
|  | Because they do not feel well |
|  | Because they find out their recent sexual partner had HIV |
|  | They find out that their sexual partner has other partners |
|  | Their partner insists |
|  | Because their friends also test |
|  | Because they have to for an insurance or medical aid or funeral policy |
|  | A healthcare worker in the community advised them to |
|  | They need to for VMMC (VOLUNTARY CIRCUMCISION IN HEALTH CLINIC) |
|  | Their mother encourages them to test |
|  | Their father encourages them to test |
|  | Their sister encourages them to test |
|  | Their brother encourages them to test |
|  | Their grandmother encourages them to test |
|  | Their grandfather encourages them to test |
|  | They test without agreeing |
|  | They get offered testing when they go to the clinic for other reasons |
|  | Because healthcare workers set up testing locations in places where they are that day |
|  | After they have sex without a condom |
|  | They were given a self-test kit |
|  | Other (Specify) |

ASK TO MEN WHO HAVE PREVIOUSLY TESTED FOR HIV ONLY (1 at S8a or 1 at S8c)

capture numeric value

D11. How many times have you seriously thought about, planned or tried to go for HIV testing but then did not test?

| _______ | # of attempts |
| --- | --- |

ASK D12 IF D11=1 OR MORE (HAVE DONE SOME ATTEMPTS). IF D11=0 SKIP TO D13

multiple response

Select all which apply / PROBE FOR AS MANY RESPONSES AS POSSIBLE

Do not read out list

D12. Why did your prior attempts to get tested for HIV fail?

| 1 | Changed mind; |
| --- | --- |
| 2 | Too scared to go through with it |
| 3 | Someone convinced me not to go through with it |
| 4 | Something else came up and I didn’t have the time or resources to do it |
| 5 | The wait was too long at the clinic |
| 6 | Too many people at the clinic |
| 7 | OTHER [SPECIFY] |
| 8 | OTHER [SPECIFY] |
| 9 | OTHER [SPECIFY] |

ASK TO MEN WHO HAVE PREVIOUSLY TESTED FOR HIV ONLY (1 at S8a or 1 at S8c)

DISPLAY SHOWCARD ON SCREEN – RATING SCALE

REPEAT SCALE WORDING AS necessary

SHOW RESPONDENT SCREEN

D13. Thinking about the last time you were tested for HIV, did the healthcare worker…

options across the top

single response per line

|  | Yes |
| --- | --- |
|  | No |
|  | Can’t remember |
|  | Prefer not to answer |

options down the side

randomise list

|  | … give you good information and advice on HIV? |
| --- | --- |
|  | … show care and kindness towards you? |
|  | … make you feel your privacy and confidentiality would be respected? |
|  | … make you trust them? |
|  | …tell you to stop doing the things you enjoy? |
|  | …advise you against traditional practices like muthi? |
|  | …appear rushed? |
|  | …appear uninterested? |

ASK TO MEN WHO HAVE PREVIOUSLY TESTED FOR HIV ONLY (1 at S8a or 1 at S8c)

multiple response- MUST SELECT 2 OPTIONS EXCEPT IF CODE 18 or 19

CONTINUE PROBING FOR MORE RESPONSES, ATLEAST 2 PER RESPONDENT

Do not read out list

D14. Why do you think some men have never had a HIV test?

|  | They may have never considered it |
| --- | --- |
|  | None of their sexual partners have HIV |
|  | They do not feel at risk of HIV |
|  | They do not have any symptoms of HIV |
|  | They prefer to go to a sangoma or prophet for health issues |
|  | They don’t know where to go for a test |
|  | They live too far from the testing clinic |
|  | They are scared of the result |
|  | It would be impossible to keep it a secret |
|  | It would make their partner think they have been unfaithful |
|  | They don’t want to have to listen to lectures from the healthcare worker |
|  | Testing is too inconvenient for them |
|  | They think it is better not to know |
|  | Somebody might see them at the testing centre |
|  | Other 1 – specify |
|  | Other 2 – specify |
|  | Other 3 – specify |
|  | Don’t know |
|  | Prefer not to answer |

ASK TO MEN WHO HAVE PREVIOUSLY TESTED FOR HIV ONLY (1 at S8a or 1 at S8c)

DISPLAY SHOWCARD ON SCREEN – RATING SCALE

REPEAT SCALE WORDING AS necessary

SHOW RESPONDENT SCREEN

D16. What is your view on the following ways of testing for HIV?

options across the top

single response per line

|  | A very bad idea |
| --- | --- |
|  | A bad idea |
|  | Neutral |
|  | A good idea |
|  | A very good idea |
|  | I don’t know |
|  | Prefer not to answer |

options down the side

randomise list

|  | Testing on your own with a kit which uses blood |
| --- | --- |
|  | Testing for HIV through sangomas |
|  | Offering testing for HIV at taverns/ shebeens |
|  | Offering testing for HIV through the workplace |
|  | Testing on your own with a kit which uses saliva |
|  | Testing at a truck stop |
|  | Testing at a taxi rank or community gathering place |
|  | Testing at a soccer game or sporting event |

ASK TO MEN WHO HAVE PREVIOUSLY TESTED FOR HIV ONLY (1 at S8a or 1 at S8c)

SINGLE RESPONSE

Do not read out list

D17. Do you know if any of your friends have been tested for HIV?

|  | I don’t know |
| --- | --- |
|  | None of them |
|  | Yes – a couple |
|  | Yes – many |
|  | Yes – most of them |
|  | Prefer not to answer |

ASK TO MEN WHO HAVE PREVIOUSLY TESTED FOR HIV ONLY (1 at S8a or 1 at S8c)

SINGLE RESPONSE

DISPLAY SHOWCARD ON SCREEN – RATING SCALE

REPEAT SCALE WORDING AS necessary

SHOW RESPONDENT SCREEN

D18. Please tell me how frequently you talk to other men to encourage them to test for their HIV status also. Rate this using a 5-point scale, where 1 means ‘Never’, 3 means ‘Sometimes’ and 5 means ‘Often’. (Select one)

| Never | Rarely | Sometimes | Regularly | Often |
| --- | --- | --- | --- | --- |
| 1 | 2 | 3 | 4 | 5 |

SECTION E: Testing History and attitudes towards testing (Never tested)

ASK TO MEN WHO HAVE NEVER TESTED FOR HIV ONLY (2 at S8a or 2 at S8c)

READ OUT: In this next section I specifically want to talk about HIV testing.

ROTATE ORDER OF E1 AND E2

ASK TO MEN WHO HAVE NEVER TESTED FOR HIV ONLY (2 at S8a or 2 at S8c)

CAPTURE OPEN ENDED ANSWER IN ENGLISH

PLEASE PROBE

E1a. What, if anything, BAD have you heard about visiting clinics for HIV testing?

What else?

|  |
| --- |

ASK TO MEN WHO HAVE NEVER TESTED FOR HIV ONLY (2 at S8a or 2 at S8c)

CAPTURE OPEN ENDED ANSWER IN ENGLISH

PLEASE PROBE

E1b. What, if anything, BAD have you heard about HIV testing outside of clinics?

What else?

|  |
| --- |

ASK TO MEN WHO HAVE NEVER TESTED FOR HIV ONLY (2 at S8a or 2 at S8c)

CAPTURE OPEN ENDED ANSWER IN ENGLISH

PLEASE PROBE

E2a. What, if anything, GOOD have you heard about visiting clinics for HIV testing?

What else?

|  |
| --- |

ASK TO MEN WHO HAVE NEVER TESTED FOR HIV ONLY (2 at S8a or 2 at S8c)

CAPTURE OPEN ENDED ANSWER IN ENGLISH

PLEASE PROBE

E2b. What, if anything, GOOD have you heard about HIV testing outside of clinics?

What else?

|  |
| --- |

ASK TO MEN WHO HAVE NEVER TESTED FOR HIV ONLY (2 at S8a or 2 at S8c)

UP TO 2 ANSWERS ALLOWED EXCEPT CODE 9 AND CODE 10 WHICH ARE MUTUALLY EXCLUSIVE

DISPLAY SHOWCARD ON SCREEN – oPTION LIST

RANDOMISE

SHOW RESPONDENT SCREEN

E4. What, if anything, is the benefit to having a HIV test? Please select up to your top 2 options from the list below

|  | Knowing HIV status helps me be safe |
| --- | --- |
|  | Knowing HIV status helps me control my health |
|  | Testing for HIV is the smart thing to do |
|  | Knowing my status means that I can be successful |
|  | Knowing my status means I can continue to enjoy life without getting sick |
|  | Knowing my status helps me enjoy life without worry |
|  | Knowing my status builds trust with my partner |
|  | Testing for my status helps me keep my family happy |
|  | Don’t know |
|  | There are no benefits |

ASK TO MEN WHO HAVE NEVER TESTED FOR HIV ONLY (2 at S8a or 2 at S8c)

DISPLAY SHOWCARD ON SCREEN – RATING SCALE

REPEAT SCALE WORDING AS necessary

SHOW RESPONDENT SCREEN

E5. I am going to read you some statements about HIV testing and would like you to tell me how much you disagree or agree with them. Please use a 5 point scale where 5 means strongly agree, 4 means agree, 3 means neither agree nor disagree, 2 means disagree and 1 means strongly disagree:

options across the top

single response per row

|  | Strongly disagree |
| --- | --- |
|  | Disagree |
|  | Neither agree nor disagree |
|  | Agree |
|  | Strongly agree |
|  | Prefer not to answer |

options down the side

randomise list

|  | I know who to turn to if I test positive for HIV |
| --- | --- |
|  | I understand the language healthcare providers use about HIV |
|  | I would feel more comfortable testing with my friends rather than on my own |
|  | I would feel more comfortable testing with my partner rather than on my own |
|  | I would rather test away from where I stay |
|  | If all my friends were going to test, then I would test too |
|  | I wouldn’t want to test incase I was HIV positive |
|  | I am worried that I will no longer be me if I find out I am HIV positive |
|  | I am worried I will die if I find out I am HIV positive |
|  | It is better to know if I am HIV positive than not to know |
|  | I would test if my partner wanted me to |
|  | I would test if the clinics were open at more convenient times |
|  | I would test if I could guarantee nobody would see me at the clinic |
|  | I do not have the time to test for HIV |
|  | I know I should test but often forget |
|  | I would test if I knew that my results would be kept private. |
|  | I would test at a clinic specifically for men |
|  | I would test at a tavern |
|  | I would test in a mall |
|  | I would test at a taxi rank |
|  | I wouldn’t test if they used a needle |
|  | Testing for HIV is a very difficult experience |
|  | After testing negative, I may feel my risk is less |

ASK TO MEN WHO HAVE NEVER TESTED FOR HIV ONLY (2 at S8a or 2 at S8c)

multiple response

Select all which apply

Do not read out list

E7. Aside from wanting to know their HIV status, what other reasons are there why men go for HIV tests?

|  | Some go as routine |
| --- | --- |
|  | After they have sex without a condom |
|  | Because they do not feel well |
|  | Because they find out their recent sexual partner had HIV |
|  | They find out that their sexual partner has other partners |
|  | Their partner insists |
|  | Because their friends also test |
|  | Because they have to for an insurance or medical aid or funeral policy |
|  | A healthcare worker in the community advised them to |
|  | They need to for VMMC (VOLUNTARY CIRCUMCISION IN HEALTH CLINIC) |
|  | Their mother encourages them to test |
|  | Their father encourages them to test |
|  | Their sister encourages them to test |
|  | Their brother encourages them to test |
|  | Their grandmother encourages them to test |
|  | Their grandfather encourages them to test |
|  | They test without agreeing |
|  | They get offered testing when they go to the clinic for other reasons |
|  | Because healthcare workers set up testing locations in places where they are that day |
|  | They were given a self test kit |
|  | Their sex partner looked unwell |
|  | OTHER SPECIFY |

ASK TO MEN WHO HAVE NEVER TESTED FOR HIV ONLY (2 at S8a or 2 at S8c)

SINGLE ANSWER

CAPTURE SPONTANEOUS ANSWER

E6. Have you ever considered going for a HIV test?

|  | Yes |
| --- | --- |
|  | No |
|  | Prefer not to answer |

ASK TO MEN WHO HAVE NEVER TESTED FOR HIV ONLY (2 at S8a or 2 at S8c)

capture numeric value

E8. How many times have you seriously thought about, planned or tried to go for HIV testing but then did not test?

| _______ | # of attempts |
| --- | --- |

ASK E9 IF E8=1 OR MORE (HAVE DONE SOME ATTEMPTS). IF E13=0 SKIP TO E10

multiple response

Select all which apply / PROBE FOR AS MANY RESPONSES AS POSSIBLE

Do not read out list

E9. Why did your prior attempts to get tested for HIV fail?

| 1 | Changed mind; not certain enough |
| --- | --- |
| 2 | Too scared to go through with it |
| 3 | Someone convinced me not to go through with it |
| 4 | Something else came up and I didn’t have the time or resources to do it |
| 5 | The wait was too long at the clinic |
| 6 | Too many people at the clinic |
| 7 | OTHER [SPECIFY] |
| 8 | OTHER [SPECIFY] |
| 9 | OTHER [SPECIFY] |

ASK TO MEN WHO HAVE NEVER TESTED FOR HIV ONLY (2 at S8a or 2 at S8c)

multiple response- MUST SELECT 2 OPTIONS EXCEPT IF CODE 18 or 19

CONTINUE PROBING FOR MORE RESPONSES, ATLEAST 2 PER RESPONDENT

Do not read out list

E10. Why have you never had a HIV test?

|  | Never considered it |
| --- | --- |
|  | None of my sexual partners have had HIV |
|  | I don’t feel at risk of HIV |
|  | I have never had any symptoms |
|  | I prefer to go to a sangoma or prophet for health issues |
|  | I don’t know where to go for a test |
|  | The testing clinic is too far away |
|  | I’m scared of the result |
|  | It would be impossible to keep it a secret |
|  | It would make my partner think I have been unfaithful |
|  | I don’t want to have to listen to lectures from the healthcare worker |
|  | Testing is too inconvenient |
|  | It is better not to know |
|  | Somebody might see me at the testing centre |
|  | Other 1 – specify |
|  | Other 2 – specify |
|  | Other 3 – specify |
|  | Don’t know |
|  | Prefer not to answer |

ASK TO MEN WHO HAVE NEVER TESTED FOR HIV ONLY (2 at S8a or 2 at S8c)

multiple response

Do not read out list

E11. Under what circumstances do you think you would consider having an HIV test?

|  | I can’t imagine any circumstances where I would have an HIV test |
| --- | --- |
|  | If one of my sexual partners was diagnosed with HIV |
|  | If one of my friends was diagnosed with HIV |
|  | If I started to feel unwell |
|  | If I started to have sexual problems |
|  | If I found out that my sex partner had other partners |
|  | If my partner insisted I had a test |
|  | If my friends decided to get tested |
|  | If I needed it for an insurance or medical aid or funeral policy |
|  | If I knew there was no chance anyone would find out |
|  | If it was more convenient |
|  | If I could more easily get to a clinic far away where no one would know me |
|  | Other 1 – specify |
|  | Other 2 – specify |
|  | Other 3 – specify |
|  | Prefer not to answer |

ASK TO MEN WHO HAVE NEVER TESTED FOR HIV ONLY (2 at S8a or 2 at S8c)

DISPLAY SHOWCARD ON SCREEN – RATING SCALE

REPEAT SCALE WORDING AS necessary

SHOW RESPONDENT SCREEN

E12. What is your view on the following ways of testing for HIV?

options across the top

single response per line

|  | A very bad idea |
| --- | --- |
|  | A bad idea |
|  | Neutral |
|  | A good idea |
|  | A very good idea |
|  | I don’t know |
|  | Prefer not to answer |

options down the side

randomise list

|  | Testing on your own with a kit which uses blood |
| --- | --- |
|  | Testing for HIV through sangomas |
|  | Offering testing for HIV at taverns/ shebeens |
|  | Offering testing for HIV through the workplace |
|  | Testing on your own with a kit which uses saliva |
|  | Testing at a truck stop |
|  | Testing at a taxi rank or community gathering place |
|  | Testing at a soccer game or sporting event |

ASK TO MEN WHO HAVE NEVER TESTED FOR HIV ONLY (2 at S8a or 2 at S8c)

SINGLE RESPONSE

Do not read out list

E13. Do you know if any of your friends have tested for HIV?

|  | I don’t know |
| --- | --- |
|  | None of them |
|  | Yes – a couple |
|  | Yes – about half |
|  | Yes – most of them |
|  | Prefer not to answer |

ASK TO MEN WHO HAVE NEVER TESTED FOR HIV ONLY (2 at S8a or 2 at S8c)

SINGLE RESPONSE

DISPLAY SHOWCARD ON SCREEN – RATING SCALE

REPEAT SCALE WORDING AS necessary

SHOW RESPONDENT SCREEN

E14. Please tell me how frequently you talk to other men to encourage them to test for their HIV status also. Rate this using a 5-point scale, where 1 means ‘Never’, 3 means ‘Sometimes’ and 5 means ‘Always’. (Select one)

| Never | Rarely | Sometimes | Regularly | Always |
| --- | --- | --- | --- | --- |
| 1 | 2 | 3 | 4 | 5 |

SECTION F: Linkage History and attitudes ~~(HIV positive respondents)~~

ASK TO MEN WHO CODED 1 at S9a OR 1 at S9c

PROGRAMMER TO ADD DROP DOWN OPTIONS WITH MONTH AND YEAR. MAKE SURE YEAR ISN’T BEFORE ANSWER AT S2

F1. In which month and year did you find out you were HIV positive? If you have been HIV Positive since birth, please use your birth month and year in the box below.

| Month | Year |
| --- | --- |

IF F1= S2 THEN SKIP TO H1, OTHERWISE ASK F2

- IF F2=1 “Initiated”
- IF F2=2 THEN “Not Initiated”

SINGLE RESPONSE

CAPTURE SPONTANEOUS ANSWER

F2. Have you ever taken medication known as ARVs, given to you by a medical professional to treat your HIV, even if you are not taking any currently?

| 1 | Yes | CONTINUE |
| --- | --- | --- |
| 2 | No | CONTINUE |

ASK IF CODE 1 “Yes” SELECTED AT F2

PROGRAMMER TO ADD DROP DOWN OPTIONS WITH MONTH AND YEAR. ENSURE DATE NOT BEFORE ANSWER AT F1

F3. After you were diagnosed as positive, when did you start your ARV medication?

| Month | Year |
| --- | --- |

ASK TO MEN WHO CODED 1 at S9a OR 1 at S9c

DISPLAY SHOWCARD ON SCREEN – RATING SCALE

REPEAT SCALE WORDING AS necessary

SHOW RESPONDENT SCREEN

F4. Please think back to when you were diagnosed with HIV. Please rate using the following scale, the extent to which you felt each of the following in response to being diagnosed with HIV:

options across the top

single response per row

|  | Strongly disagree |
| --- | --- |
|  | Disagree |
|  | Neither agree or disagree |
|  | Agree |
|  | Strongly agree |

options down the side

randomise list

|  | I felt that I am the one who is responsible for what has happened |
| --- | --- |
|  | I thought that I have to accept that this has happened |
|  | I thought that I have to accept the situation |
|  | I often thought about how I feel about what I have experienced |
|  | I thought of something nice instead of what had happened |
|  | I thought about how to change the situation |
|  | I thought about a plan of what I can do best |
|  | I thought I could learn something from the situation |
|  | I thought that I could become a stronger person as a result of what had happened |
|  | I thought that it hadn’t been too bad compared to other things |
|  | I told myself that there are worse things in life |
|  | I kept thinking about how terrible it was what I have experienced |
|  | I kept feeling that others are responsible for what has happened |
|  | I kept feeling that basically the cause lies with others |
|  | I wanted to hurt other people |
|  | I felt full of rage |

ASK TO MEN WHO CODED 1 at S9a OR 1 at S9c

multiple response

Do not read out list

F5. Please describe what you recall the nurse telling you once you found out you were HIV Positive

|  | That I need to see another healthcare worker |
| --- | --- |
|  | That I must no longer have sex with multiple partners |
|  | That I must always wear a condom |
|  | That I must wait for my CD4 count/baseline bloods before starting on  ARVs |
|  | If I take ARVs every day I’m less likely to infect my partner |
|  | That HIV is not a death sentence |
|  | That I can live for many years with HIV |
|  | That I must no longer use muthi/traditional medicine |
|  | That I must cut down/ not use alcohol any more |
|  | Other [SPECIFY] |
|  | Other [SPECIFY] |
|  | Does not recall |

ASK TO MEN WHO CODED 1 at S9a OR 1 at S9c

SINGLE RESPONSE

READ OUT

F6. Thinking about the nurse’s tone and demeanor, which one of the following best describes how he/she spoke to you?

| 1 | The nurse was caring towards me |
| --- | --- |
| 2 | The nurse was arrogant to me |
| 3 | The nurse was in a hurry |
| 4 | The nurse was rude to me |
| 6 | The nurse was sweet to me |
| 7 | The nurse did not care about me |

ASK TO MEN WHO CODED 1 at S9a OR 1 at S9c

DISPLAY SHOWCARD ON SCREEN – RATING SCALE (DO NOT DISPLAY ‘DON’T KNOW’ ANSWER)

SINGLE RESPONSE

REPEAT SCALE WORDING AS necessary (DO NOT READ ‘DON’T KNOW’ ANSWER)

SHOW RESPONDENT SCREEN

F7. Thinking about what you know about taking ARVs for your HIV, how knowledgeable do you feel about taking ARVs?

| 1 | Not at all knowledgeable |
| --- | --- |
| 2 | Not very knowledgeable |
| 3 | Somewhat knowledgeable |
| 4 | Very knowledgeable |
| 99 | Don’t know |

ASK TO MEN WHO CODED 1 at S9a OR 1 at S9c

DISPLAY SHOWCARD ON SCREEN – RATING SCALE

REPEAT SCALE WORDING AS necessary

SHOW RESPONDENT SCREEN

F8. I am going to read you some statements about starting ARVS (HIV medicine) after finding out you are HIV positive and would like you to tell me how much you disagree or agree with them. If you are already taking ARVs, please remember how you felt before you took ARVs and answer accordingly.

options across the top

single response per row

|  | Strongly disagree |
| --- | --- |
|  | Partially disagree |
|  | Neither agree nor disagree |
|  | Partially agree |
|  | Strongly agree |
|  | Prefer not to answer |

options down the side

randomise list

|  | I do not see the point in taking ARVS |
| --- | --- |
|  | I do not trust ARVs |
|  | I do not understand how ARVs help with HIV |
|  | Only people whose CD4 counts are low should take ARVs |
|  | Only people who have symptoms of illness need to take ARVs |
|  | It is too hard to take pills every day |
|  | I hear that ARVs make people feel bad |
|  | Going to the clinic to collect ARVs is too much hassle |
|  | I am scared that people might see me collecting my ARVs |
|  | I need to hide ARVs from people in my house and it is hard to do so. |
|  | I need to hide ARVs from my friends and it is hard to do so. |
|  | It is fine to take ARVs publicly |
|  | ARVs will help me live for a long time |
|  | If I take ARVs regularly, I’m less likely to pass the virus on to others |
|  | People who take ARVs must not drink alcohol |

SECTION G: Linkage History and attitudes

ASK TO MEN WHO CODED 2 at S9a OR 2 at S9c OR “NO” AT S8C, 3 OR 4 AT S9A OR 3 AT S9C (I.E. DO NOT ASK IF HIV POSITIVE, ASK IF DON’T KNOW, NEVER TESTED OR DID NOT RECEIVE RESULTS)

READ OUT: I now want you to imagine that you have just been diagnosed with HIV. Please put yourself into that mindset and then think about how you might react

ASK TO MEN WHO CODED 2 at S9a OR 2 at S9c OR “NO” AT S8C, 3 OR 4 AT S9A OR 3 AT S9C (I.E. DO NOT ASK IF HIV POSITIVE, ASK IF DON’T KNOW, NEVER TESTED OR DID NOT RECEIVE RESULTS)

DISPLAY SHOWCARD ON SCREEN – RATING SCALE

REPEAT SCALE WORDING AS necessary

SHOW RESPONDENT SCREEN

G1. Please rate using the scale below, the extent to which you think you’d feel each of the following if you were diagnosed with HIV:

options across the top

single response per row

|  | Strongly disagree |
| --- | --- |
|  | Disagree |
|  | Neither agree or disagree |
|  | Agree |
|  | Strongly agree |

options down the side

randomise list

|  | I felt that I am the one who is responsible for what has happened |
| --- | --- |
|  | I thought that I have to accept that this has happened |
|  | I thought that I have to accept the situation |
|  | I often thought about how I feel about what I have experienced |
|  | I thought of something nice instead of what had happened |
|  | I thought about how to change the situation |
|  | I thought about a plan of what I can do best |
|  | I thought I could learn something from the situation |
|  | I thought that I could become a stronger person as a result of what had happened |
|  | I thought that it hadn’t been too bad compared to other things |
|  | I told myself that there are worse things in life |
|  | I kept thinking about how terrible it was what I have experienced |
|  | I kept feeling that others are responsible for what has happened |
|  | I kept feeling that basically the cause lies with others |
|  | I wanted to hurt other people |
|  | I felt full of rage |

SECTION H: PrEP profile and attitudes

ASK TO HIV NEGATIVE MEN ONLY WHO CODED 2/3 at S9a OR 2/3 at S9c OR IF NEVER TESTED/ DON’T KNOW STATUS

READ OUT: I now want to talk to you about a medicine in development which may be available one day. It is a pill you can take and then if you have sex with someone who is HIV Positive, you are protected from catching HIV. You should take 1 pill per day, with or without food but at a similar time each day. The pill gives you almost complete protection from HIV when you have sex without a condom but not from unwanted pregnancies or other STIs. Some people have experienced stomach problems like nausea and diarrhea when starting on this medication but usually these should go away by themselves within a few weeks. Imagine that it is free of charge for men to access.

ASK TO HIV NEGATIVE MEN ONLY WHO CODED 2/3 at S9a OR 2/3 at S9c OR IF NEVER TESTED/ DON’T KNOW STATUS

SINGLE ANSWER

CAPTURE SPONTANEOUS ANSWER

H1. Have you ever heard of such a pill?

|  | Yes |
| --- | --- |
|  | No |
|  | Can’t remember/ Don’t know |
|  | Prefer not to answer |

ASK TO HIV NEGATIVE MEN ONLY WHO CODED 2/3 at S9a OR 2/3 at S9c OR IF NEVER TESTED/ DON’T KNOW STATUS

multiple responseS EXCEPT CODE 10

DISPLAY SHOWCARD ON SCREEN – ANSWER OPTIONS

RANDOMISE OPTIONS

SHOW RESPONDENT SCREEN

H2. Which of these words best describes how you feel after what you just heard?

|  | Curious |
| --- | --- |
|  | Happy |
|  | Skeptical |
|  | Bored |
|  | Hopeful |
|  | Interested |
|  | Excited |
|  | Anxious |
|  | Scared |
|  | None of the above |

ASK TO HIV NEGATIVE MEN ONLY WHO CODED 2/3 at S9a OR 2/3 at S9c OR IF NEVER TESTED/ DON’T KNOW STATUS

SINGLE RESPONSE

DISPLAY SHOWCARD ON SCREEN – RATING SCALE

REPEAT SCALE WORDING AS necessary

SHOW RESPONDENT SCREEN

H3. On a scale of 1-5 where 1 is Not at all likely, 2 is not likely, 3 is unsure, 4 is likely and 5 is very likely, how keen would you be to take this pill every day to prevent HIV?

| Not at all likely | Not likely | Unsure | Likely | Very  Likely |
| --- | --- | --- | --- | --- |
| 1 | 2 | 3 | 4 | 5 |

ASK TO HIV NEGATIVE MEN ONLY WHO CODED 2/3 at S9a OR 2/3 at S9c OR IF NEVER TESTED/ DON’T KNOW STATUS

multiple responseS NOT LESS THAN 2, NOT MORE THAN 4 ANSWERS

DISPLAY SHOWCARD ON SCREEN – RATING SCALE

RANDOMISE

SHOW RESPONDENT SCREEN

H4. Now we would like to ask you how would you ideally want to feel if you were to take this pill. Which of the following is most relevant to you? Please choose not less than 2 but not more than 4 answers.

| 1 | To feel secure and not concerned about the future |
| --- | --- |
| 2 | To return comfort and harmony |
| 3 | To maintain a negative status |
| 4 | To be certain that I will be able to do everything I need |
| 5 | To feel that I’m well-educated in health matters |
| 6 | To feel that I am making a thought-through choice |
| 7 | To feel that I choose the best medications |
| 8 | To feel that I can achieve success without any obstacles |
| 9 | To have energy to do what I want |
| 10 | To feel invigorated and be ready for new opportunities |
| 11 | To enjoy life without thinking about consequences |
| 12 | To be carefree |
| 13 | To fully enjoy the company of other people |
| 14 | To feel free and easy in any company |
| 15 | To bring joy to my family |
| 16 | To be a support for my family |

ASK TO HIV NEGATIVE MEN ONLY WHO CODED 2/3 at S9a OR 2/3 at S9c OR IF NEVER TESTED/ DON’T KNOW STATUS

SINGLE RESPONSE

DISPLAY SHOWCARD ON SCREEN – RATING SCALE

REPEAT SCALE WORDING AS necessary

SHOW RESPONDENT SCREEN

H5. Now imagine that you would not need to take a pill every day, but would take 2 pills 2-24 hours before sex, 1 more pill after 24 hours, and 1 more pill another 24 hours after that. You previously said that you were/ would have been [INSERT ANSWER FROM H3] to take the daily pill. How does your answer change, if at all?

|  | Less likely to take the pill |
| --- | --- |
|  | Stays the same |
|  | More likely to take the pill |

ASK TO HIV NEGATIVE MEN ONLY WHO CODED 2/3 at S9a OR 2/3 at S9c OR IF NEVER TESTED/ DON’T KNOW STATUS

SINGLE RESPONSE

DISPLAY SHOWCARD ON SCREEN – RATING SCALE

REPEAT SCALE WORDING AS necessary

SHOW RESPONDENT SCREEN

H6. Now imagine that you would take the pill for 4 days a week instead of every day. You previously said that you were/ would have been [INSERT ANSWER FROM H3] to take the daily pill. How does your answer change, if at all?

|  | Less likely to take the pill |
| --- | --- |
|  | Stays the same |
|  | More likely to take the pill |

ASK TO HIV NEGATIVE MEN ONLY WHO CODED 2/3 at S9a OR 2/3 at S9c OR IF NEVER TESTED/ DON’T KNOW STATUS

SINGLE RESPONSE

DISPLAY SHOWCARD ON SCREEN – RATING SCALE

REPEAT SCALE WORDING AS necessary

SHOW RESPONDENT SCREEN

H7. Now imagine that you would take the pill every day only during periods where you know you will have more sex such as December holidays rather than all year. You previously said that you were/ would have been [INSERT ANSWER FROM H3] to take the daily pill. How does your answer change, if at all?

|  | Less likely to take the pill |
| --- | --- |
|  | Stays the same |
|  | More likely to take the pill |

ASK TO HIV NEGATIVE MEN ONLY WHO CODED 2/3 at S9a OR 2/3 at S9c OR IF NEVER TESTED/ DON’T KNOW STATUS

CAPTURE NUMERIC ENTRY

RANGE 0 – 99999 ZAR

H8a. Imagine you would need to buy the pills every month. How much would you be willing to pay a month?

| …..ZAR per month |
| --- |

ASK TO HIV NEGATIVE MEN ONLY WHO CODED 2/3 at S9a OR 2/3 at S9c OR IF NEVER TESTED/ DON’T KNOW STATUS

PROGRAMMER: PICK A RANDOM PRICE POINT, IF RESPONDENT SELECTS T2B THEN MOVE UP TO THE NEXT PRICE POINT, IF NOT T2B THEN MOVE DOWN A PRICE POINT UNTIL A T2B OPTION IS SELECTED

H8b. Willingness to pay

Imagine it cost [150ZAR, 200ZAR, 250ZAR, 300ZAR, 350ZAR] per month for the daily pills. How willing would you be to buy the pills at this price?

|  | Definitely would not buy |
| --- | --- |
|  | Probably would not buy |
|  | Not sure |
|  | Probably would buy |
|  | Definitely would buy |

ASK TO HIV NEGATIVE MEN ONLY WHO CODED 2/3 at S9a OR 2/3 at S9c OR IF NEVER TESTED/ DON’T KNOW STATUS

DISPLAY SHOWCARD ON SCREEN – RATING SCALE

REPEAT SCALE WORDING AS necessary

SHOW RESPONDENT SCREEN

H9. I am going to read you some statements about the pill and would like you to tell me how much you disagree or agree with them. Please use a 5 point scale where 5 means strongly agree, 4 means slightly agree, 3 means neither agree nor disagree, 2 means slightly disagree and 1 means strongly disagree:

options across the top

single response per row

|  | Strongly disagree |
| --- | --- |
|  | Slightly disagree |
|  | Neither agree nor disagree |
|  | Slightly agree |
|  | Strongly agree |
|  | Prefer not to answer |

options down the side

randomise list

|  | Some men like me would find it difficult to remember to take a pill every day |
| --- | --- |
|  | Some men like me would need to hide the pill from a partner incase he/she accused him of of cheating |
|  | Some men like me would find it hard to hide the pills from others |
|  | I feel like I would need to keep it secret from my partner that I am taking the pill |
|  | I feel like I would need to keep it secret from my friends that I am taking the pill |
|  | I feel like I would need to keep it secret from my family that I am taking the pill |
|  | Taking pills is for women |
|  | Men like me would stop taking the pill if it made them feel bad |
|  | Men like me would not go to a clinic to collect pills |
|  | Some men might sell their pills to drug users |
|  | I could fit taking pills into my daily routine easily |

ASK TO HIV NEGATIVE MEN ONLY WHO CODED 2/3 at S9a OR 2/3 at S9c OR IF NEVER TESTED/ DON’T KNOW STATUS

SINGLE RESPONSE

DISPLAY SHOWCARD ON SCREEN – RATING SCALE

REPEAT SCALE WORDING AS necessary

SHOW RESPONDENT SCREEN

H10. Now imagine that every time you went to collect new pills (e.g. every 3 months) you had to have a HIV test to make sure you were still negative. You previously said that you were/ would have been [INSERT ANSWER FROM H3] to take the daily pill. How does your answer change, if at all?

|  | Less likely to take the pill |
| --- | --- |
|  | Stays the same |
|  | More likely to take the pill |

ASK TO HIV NEGATIVE MEN ONLY WHO CODED 2/3 at S9a OR 2/3 at S9c OR IF NEVER TESTED/ DON’T KNOW STATUS

SINGLE ANSWER

CAPTURE SPONTANEOUS ANSWER

H11. There is another pill that some people take for 28 days after they have sex without a condom and are worried about HIV. This is sometimes called PEP. Have you ever used PEP?

|  | Yes |
| --- | --- |
|  | No |

SECTION H: Demographics

ASK ALL

SINGLE RESPONSE PER STATEMENT

COMPLETE BY OBSERVATION WHERE POSSIBLE

H1. Now I would like to read out a list of things that will help us understand a bit more about your lifestyle. Please tell me which of the following are presently in your household?

|  |  | Yes | No |
| --- | --- | --- | --- |
| 1 | Hot running water from a geyser | 1 | 2 |
| 2 | Computer – Desktop / Laptop | 1 | 2 |
| 3 | Electric Stove | 1 | 2 |
| 4 | Do you employ a domestic worker in this household (by this we mean a live-in or part-time domestics and/or gardeners)? | 1 | 2 |
| 5 | 0 or 1 radio set in household | 1 | 2 |
| 6 | Is there a Flush toilet inside or outside house | 1 | 2 |
| 7 | Do you or anyone who lives in this household have a motor vehicle/s i.e. car, van, bakkie, truck, lorry etc. | 1 | 2 |
| 8 | Washing machine | 1 | 2 |
| 9 | Refrigerator or combined fridge / freezer | 1 | 2 |
| 10 | Vacuum cleaner / floor polisher | 1 | 2 |
| 11 | Pay TV such as M-Net / DStv / Starsat subscription | 1 | 2 |
| 12 | Dishwashing machine | 1 | 2 |
| 13 | 3 or more cell phones in household | 1 | 2 |
| 14 | 2 cell phones in household | 1 | 2 |
| 15 | Home security service | 1 | 2 |
| 16 | Deep freezer – free standing | 1 | 2 |
| 17 | Microwave oven | 1 | 2 |
| 18 | Do you live in a rural area | 1 | 2 |
| 19 | Interviewer to observe and select yes or no. Do not ask respondent:  Does respondent live in either a house, cluster house (flat or semi- detached dwelling) or a townhouse? | 1 | 2 |
| 20 | DVD player / Blu-ray Player | 1 | 2 |
| 21 | Tumble Dryer | 1 | 2 |
| 22 | Home theatre system | 1 | 2 |
| 23 | Home telephone (this can be a Telkom or Neotel landline but not a cellphone) | 1 | 2 |
| 24 | Swimming pool | 1 | 2 |
| 25 | Tap water in house / on plot | 1 | 2 |
| 26 | Built-in kitchen sink | 1 | 2 |
| 27 | TV/s | 1 | 2 |
| 28 | Air conditioner: by air-conditioner I am referring to a major appliance or system designed to change the air temperature and humidity in an area. It is not a fan or a water cooler. | 1 | 2 |
| 29 | Metropolitan dweller (250 000+) | 1 | 2 |

H1.2. Do you have the capability to use WhatsApp or Facebook on your mobile phone?

| 1 | Yes |
| --- | --- |
| 2 | No |
| 3 | Not sure |

H1.3. Do you ever buy data for your mobile phone?

| 1 | Yes |
| --- | --- |
| 2 | No |

SECTION I: Self Testing (ASK TO HIV NEGATIVE MEN ONLY WHO CODED 2/3 at S9a OR 2/3 at S9c) OR IF NEVER TESTED/ DON’T KNOW STATUS

Thank you for your participation in this research- we very much appreciate your answers.

There is a final part of the study, which you may participate in if you choose, about HIV self-testing. Is it OK if I explain that to you now? [ONLY CONTINUE IF RESPONDENT SAYS YES]

| Continue | YES □ NO □ |
| --- | --- |

READ SCREENER WITH RESPONDENT AND THEN HAND OVER FOR FINAL CONSENT

What we are doing

We are inviting everyone who completed the survey to complete an optional self-test for HIV and report the answers back to us. The reason why we want to do this is to find out how people’s attitudes and experiences differ according to their HIV status.

Your participation

If you decide to take part, this is what will happen:

- I will give you an HIV self-testing kit and explain to you how it works. We will watch a video together to explain this, and I can send you the video if you would like to watch it at another time. I will also give you some written materials to explain how to do the test, and what to do after finding out the result
- We would like you to send us the results of your test, although you do not have to do this if you don’t want to. To allow you to send the results back, I will help you download an app onto your mobile phone so you can do this safely and securely
- I will then give you a number, which is unique to you, that you should enter into the app. This is so you can provide your results without telling us your name or giving your phone number.
- You will then complete the self-test in your own time and send us the results using the app.
- Through the app you will receive information about what to do next if the result is positive.
- The time required to give you the required information today will be approximately 10 minutes, and a further 15 minutes will be required to do the test and report the results. To reimburse you for any airtime you use during this process, we will give you airtime worth ZAR 29-35 (check table below) only after you have sent us the results (within 48 hours).)


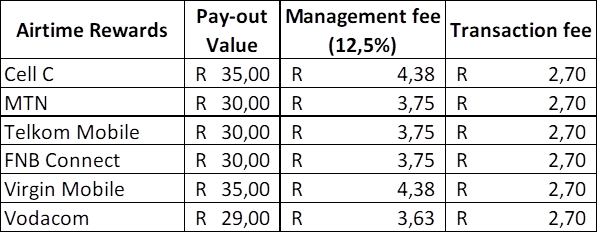


Confidentiality

Your HIV status will be kept on an encrypted server (i.e. on a secure computer) and will not be available to others and will be kept confidential to the extent possible by law. Your HIV status may be reviewed by people responsible for making sure that research is done properly, including members of the ethics committee at FDP. (All of these people are required to keep your identity confidential.) Otherwise, your HIV status will be available only to people working on the study, but it will never be linked to any information that could be identify you (such as name or telephone number). Your name will only appear on this consent document and will only be seen by people working on this study. Your telephone number will only be used to transfer you airtime.

All future use of the stored data will be subject to further Research Ethics Committee review and approval.

The testing data you provide will be stored in an anonymous format for up to two years by Ipsos and PSI. After that time it will be destroyed.

The app on your mobile phone will contain the data that you provide to us (including your HIV status) but we strongly suggest you delete the app from your phone after providing answers.

Risks

The most significant risk is a breach of confidentiality, but we have put measures in place to ensure that this does not happen.

We realise that this is sensitive topic, so you may refuse to take the test if you don’t want to.

Benefits

It may be beneficial for you to know your HIV status so that you can access treatment, if required.

Who to contact if you have been harmed or have any concerns

This research has been approved by the FDP Research Ethics Committee and PSI’s REC. If you have any complaints about ethical aspects of the research or feel that you have been harmed in any way by participating in this study, please call +27 (0) 12 816 9000 or email foundation@foundation.co.za

If you have concerns or questions about the research you may call the project leader Jean Moolman, +27 12 428 7400, Jean.Moolman@askafrika.co.za.

HAND OVER TABLET

| The research has been explained to me and I have been given a full explanation about the possible uses of any personally identifying information | | YES □ NO □  IF “No” then skip to end |
| --- | --- | --- |
| I consent to participating in this research | | YES □ NO □  IF “No” then skip to end |
| I consent to the use of my phone number to contact me (by phone or whatsapp) so that I can report the results of my test | | YES □ NO □ |
| Print full name |  | |
| Signature |  | |
| Date |  | |

THANK YOU. PLEASE HAND SCREEN BACK TO INTERVIEWER

INTERVIEWER: NOW FOLLOW PROCEDURE FOR EXPLAINING TEST KIT. REMEMBER TO INSERT ID NUMBER INTO APP

INTERVIEW QUALITY QUESTIONS

INTERVIEWER: NOW FOLLOW PROCEDURE FOR EXPLAINING TEST KIT. REMEMBER TO INSERT ID NUMBER INTO APP

OPEN VERBATIM: INTERVIEWER CAPTURE ANY INTERVIEW COMMENTS.
