# Supplementary material for: Targeting interventions for HIV testing and treatment uptake: An attitudinal and behavioural segmentation of men aged 20–34 in KwaZulu-Natal and Mpumalanga, South Africa
Source: PLoS One. 2021 Mar 10;16(3):e0247483. doi: 10.1371/journal.pone.0247483 (PMC7946194; doi:10.1371/journal.pone.0247483)
Supplement: S1 Table — (DOCX) [file pone.0247483.s003.docx]

**List of segmentation variables.** Question numbers refer to questionnaire also included in supplementary materials.

| A3 | I am going to read you some statements and would like you to tell me how much you disagree or agree with them? |
| --- | --- |
| A15 | I am going to read you some statements and would like you to tell me how much you disagree or agree with them? |
| B1 | *I’m going to list several personality traits which may apply or may not apply to you. Please think about how your friends might describe you and how much they would agree or disagree with the following personality traits* |
| B2 | From this list, which is the MOST like you and which is the LEAST like you? |
| B3 | I am now going to list several statements about different outlooks on life. Please think about how much you agree or disagree with the following statements and how closely they reflect your outlook on life |
| B4 | Which two of these statements best describes your outlook on life? |
| B5 | I’m now going to read out some statements. For each, please let me know the extent you agree or disagree |
| B6 | When thinking about relationships between men and women, to what extent do you agree or disagree with the following statements? |
| B7 | I now want to ask some sensitive questions about having sex. To the best of your memory, how old were you when you first had sex? |
| B8 | How many different sexual partners have you had in the past 12 months? |
| B9 | How many people are you currently having regular sex with? |
| B14A | How regularly, if at all, did you wear a condom with them? |
| B15 | How easy do you find it to talk about sexual health with each of the following people? For example, using condoms, protecting from HIV, protecting from other sexually transmitted infections etc. |
| B17 | Please indicate to what extend you agree or disagree with the following. Please be as honest and accurate as you can throughout. |
| C1 | Generally, how would you describe your state of health? |
| C2 | Over the last 2 weeks, how often have you experienced any of the following problems? |
| C5 | How frequently, if at all, do you visit a healthcare clinic? |
| C9 | How much do you agree or disagree with the following statements? |
| C10 | I’m going to start with a series of statements about HIV and I’d like you to tell me if you think they are true or false |
| C11 | I’m going to read several statements about HIV. After I read each of them, please tell me how much you agree or disagree with each of them by rating your level of agreement with the statement |
| C12 | For the next few questions we’d like you to just give us your best guess at the answer. No one knows the answer to these questions for sure so there is no right or wrong answer. Just tell us what you think. At what age do you think most men and most women get HIV? |
| C13A | Out of 100 men within your age group, how many do you think have HIV in your local community? |
| C13B | Out of 100 women within your age group, how many do you think have HIV in your local community? |
| D4 / E4 | What, if anything, is the benefit to having a HIV test? Please select up to your top 2 options from the list below |
| D9 / E7 | Aside from wanting to know their HIV status, what other reasons are there why men go for HIV tests? |
| D11 / E8 | How many times have you seriously thought about, planned or tried to go for HIV testing but then did not test? |
| D14 / E10 | Why do you think some men have never had a HIV test? |
| D16 / E12 | What is your view on the following ways of testing for HIV? |
| F4 / G1 | Please rate using the following scale, the extent to which you felt each of the following in response to being diagnosed with HIV |
